# Adaptive Context Caching for IoT-Based Applications: A Reinforcement Learning Approach

**DOI:** 10.3390/s23104767

**Published:** 2023-05-15

**Authors:** Shakthi Weerasinghe, Arkady Zaslavsky, Seng Wai Loke, Alireza Hassani, Alexey Medvedev, Amin Abken

**Affiliations:** School of Information Technology, Deakin University, Geelong, VIC 3145, Australia

**Keywords:** internet of things, context-awareness, adaptive context caching, reinforcement learning

## Abstract

Making internet-of-things (IoT)-based applications context-aware demands large amounts of raw data to be collected, interpreted, stored, and reused or repurposed if needed from many domains and applications. Context is transient but interpreted data can be distinguished from IoT data in many aspects. Managing context in cache is a novel area of research that has been given very little attention. Performance metric-driven adaptive context caching (ACOCA) can have a profound impact on the performance and cost efficiency of context-management platforms (CMPs) when responding to context queries in realtime. Our paper proposes an ACOCA mechanism to maximize both the cost and performance efficiency of a CMP in near realtime. Our novel mechanism encompasses the entire context-management life cycle. This, in turn, distinctively addresses the problems of efficiently selecting context for caching and managing the additional costs of context management in the cache. We demonstrate that our mechanism results in long-term efficiencies for the CMP that have not been observed in any previous study. The mechanism employs a novel, scalable, and selective context-caching agent implemented using the twin delayed deep deterministic policy gradient method. It further incorporates an adaptive context-refresh switching policy, a time-aware eviction policy, and a latent caching decision management policy. We point out in our findings that the additional complexity of adaptation introduced to the CMP through ACOCA is significantly justified, considering the cost and performance gains achieved. Our algorithm is evaluated using a real-world inspired heterogeneous context-query load and a data set based on parking-related traffic in Melbourne, Australia. This paper presents and benchmarks the proposed scheme against traditional and context-aware caching policies. We demonstrate that ACOCA outperforms the benchmarks in both cost and performance efficiency, i.e., up to 68.6%, 84.7%, and 67% more cost efficient compared to traditional data caching policies to cache context, redirector mode, and context-aware adaptive data caching under real-world-like circumstances.

## 1. Introduction

The Internet of Things (IoT) has significantly grown in the last decade and the experts refer to IoT as a source of big data [1]. Applications based on data generated from the IoT can benefit immensely from the variety and volume of the big IoT data to infer context information and facilitate context-aware applications [2]. By the definition of Abowd et al. [2], context is “any information that can be used to characterize the situation of an entity. Any entity is a person, place, or object that is considered relevant to the interaction between a user and an application, including the user and the applications themselves”. Therefore, in an environment subject to constant interactions between many different entities, context acts as metainformation for an application to perform more relevantly (i.e., context-aware), e.g., as per the current situation of the user. For example, a mobile application may automatically suggest the user take a train instead of the regular bus (based on historical travel-activity data and relevant context) to arrive on time for a scheduled meeting in the calendar. The application may trigger this suggestion in the event of leaving the house and identifying the user as being “stressed” (i.e., context about the user). The application is thus imposing as if it has a cognitive sense about the user.

Context is fundamentally different from “data” traditionally discussed in the literature, e.g., flat files such as multimedia and database records since context information is interpreted as information about an entity or a collection of entities *using the relevant data*. As in our example, the calendar appointment, transport timetables, date and time departing the house, current and destination locations, movement of the user, and finally the users’ travel patterns are traditional “data” from which the users’ decision to travel (the current situation, i.e., the context) is a decision supported in a smart way.

### 1.1. Why Cache Context Information?

Context is transient in nature, e.g., the context of the user changes when moving closer to the destination. Similar is the context of other entities. For example, although the application suggested the train, it may be the case that the train failed to arrive on time at the destination platform due to congestion occurring later. The context of being “busy” at the destination has evolved over time. Therefore, the time criticality of responding to context queries from applications that implement features, such as in our example, is significant. Compare our example to an application that retrieves train schedules (which is purely data without context) for many users, e.g., *cancelled* trains are shown only as *delayed* in live Google Maps.

The literature points to caching the popular data [3,4,5] such as the timetables to improve the response time of the application. However, it may not reflect the changes and interruptions. Database transaction optimizers may define stored procedures and/or views to respond to such popular data queries. As much as the data retrieval process for context derivation can benefit from optimizing techniques at the producer when the context providers are essentially not IoT devices (e.g., the calendar in the above example), deriving context would still yield an overhead that minimizes this benefit such that delivered context may no longer be relevant to the entity. Note that the raw data from which the context is derived from for each context entity, originate from “relevant” context providers [6] selected from billions of possible providers for billions of context consumers [1]. Therefore, streamlining the process of managing context retrieval or inventing an innovative method to reuse and repurpose retrieved and derived context using a caching technique is a compelling research problem.

### 1.2. Problems with Caching Context Information

IoT context providers are heterogeneous in many ways, e.g., mobility (e.g., a moving train with attached sensors versus a stationary weather station), origin (e.g., sensor versus an application such as the calendar), data types and structures (e.g., JSON versus video stream), protocols (e.g., MQTT versus HTTP), etc. Context-management platforms (CMP) are middleware that derive and deliver context to context-aware applications, providing an abstract and unified view of the IoT ecosystem. Context-as-a-Service [7], Nexus [8], and FIWARE Orion [9] are such examples. Therefore, unlike approaches to traditional or context-aware data caching, caching a copy of IoT data would not be cost or performance efficient when managing context [10].

**Figure 1:**
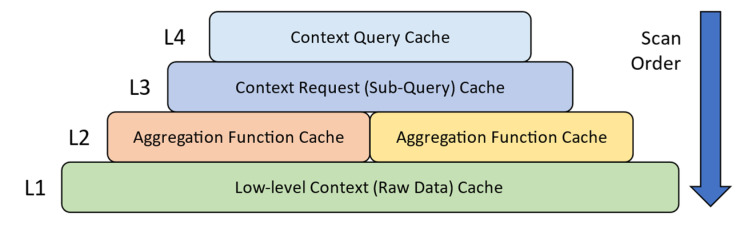
Logical hierarchy of a context cache inspired by [12].

First, context information cannot be cached based on the write-once-read-many concept, as in data caching, due to transiency. Context needs to be refreshed [11,12], similar to cached IoT data [13,14]. Refreshing incurs a recurring cost of processing and data retrieval during cache residence. An important difference between context and cached IoT data is that a refresh operation for a cached context may retrieve data from multiple context providers. A piece of cached IoT data has a single origin because the data is a copy stored in the cache. In contrast, the interpreted context is a result of several relevant data from several different sources [7]. Therefore, the cost of refreshing context information (such as about a situation) in the cache is exponentially greater than refreshing IoT data unless a piece of IoT data is directly accessed as a low-level context (e.g., temperature as context). One should also consider the processing cost involved in updating all relevant contexts at this expense. For example, Medvedev et al. [12] updated all the derived context (L2 and above) for each context refreshed in L1 of the context hierarchy, as illustrated in Figure 1. Assuming for a piece of context information, indexed as $i\in \left\{1,2,\ldots I\right\}$, where *I* is the number of all cached context information at a given point in time, data are retrieved from *M* context providers and reactively updates the C number of relevant derived context information, then the complexity of refreshing is at least $O\left(M+C\right)$ in the best case, compared to $O\left(1\right)$ of cached transient “data” [15].

Second, the validity of context is a unique problem when managing cached context information. For example, the destination’s “business” is subject to change over time. If “business” is cached, it should be updated in near realtime. However, consider the context spaces theory [16]. “Business” as a state may be true over a range of values of the context attributes used to define the context. Unlike refreshing transient data (e.g., cached IoT data), context refreshing needs not always be synchronized with the sampling rate of the data provider [17]. Refresh rate and the context-management processing overhead can be minimized using complex techniques such as those investigated in this paper and monitoring techniques. This area of research is yet to be thoroughly investigated as we do in our work also because there is very limited work done, at least in theory [18].

Third, the popularity of accessing a piece of data is often considered the context in context-aware data caching [3,5,19,20]. Note that these authors refer to the metadata about the data evaluated for caching as “context” here. “Context” referred to in this paper is rather beyond this scope and is used to describe situations of entities that are more physically recognizable in the environment. The impact of trying to maximize the hit rate (HR) to a CMP can be less significant compared to minimizing (a) the probability of delay (PD)–the probability that the response latency to a context query takes longer than agreed in the service level agreement [11], and (b) the $\bar{Gain}$–earnings deducted of all costs incurred per context-query response. For example, consider that the latency to derive a context is 200 ms (the sum of processing overhead and the retrieval latency) and is on average accessed each 1s by the consumers. If the context is cached and refreshed every 500 ms, the cached context would be twice as expensive to manage than being retrieved on demand (referred to as the redirector mode [12]). We refer to such a scenario as being retrieval inefficient, i.e., HR→1 but the gain is minimized due to the less than cost efficient number of refreshing operations. We refer to the problem of exponentially increasing the cost of adaptive context management in the cache as the “exploding cost of context management” problem. The problem is a result of ACOCA being a lifecycle process. Therefore, caching context needs to be viewed from a different perspective than data, especially for IoT-based applications that are time critical in nature which require context to be derived and delivered to the consumer fast while being inexpensive. Context-aware caching or any other traditional data-caching techniques may not be fully applicable in this setup.

**Figure 2:**
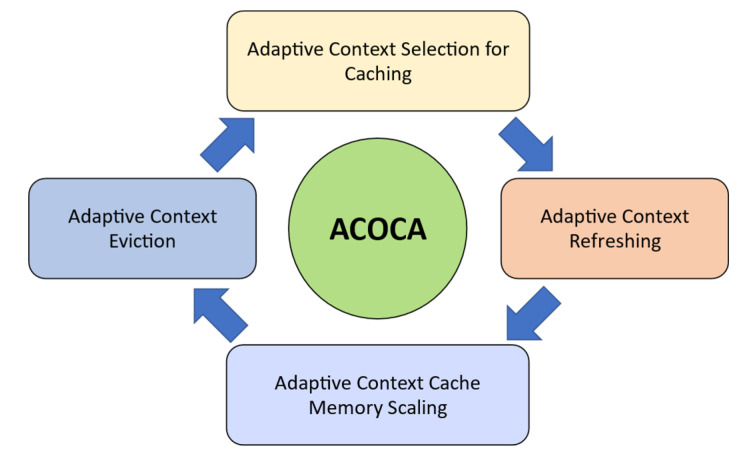
Context-cache lifecycle inspired by [15].

Figure 2 illustrates this lifecycle of cached context, each of which we need to address to satisfy both cost and performance-efficiency objectives. Costs incurred during the life cycle of a piece of context information are referred to as the lifecycle costs.

ACOCA’s context-management lifecycle contains four main stages: selection, refreshing, scaling, and eviction. First, selection involves efficiently selecting what and when to cache among billions of heterogeneous contexts. While the research problem primarily concerns selecting the context information that is expected to improve the efficiency of the CMP, it is also important that the context selection overhead is minimized, either by the number of context information being evaluated for caching, the frequency of making caching decisions and re-evaluation for caching, or reducing the complexity of the selection process (as in [21]). Second, adaptive refreshing context involves dynamically changing the refresh rate of each piece of context information to maximize freshness (i.e., validity) while minimizing cost. Third, adaptive resource scaling in ACOCA aims at maximizing the utility of context caches. In [15], we introduce the term ‘hold-up cost’ which is defined as the cost of cache memory to the CMP from context information that occupy space without producing ‘justifiable’ (subjective depending on factors such as complexity of the context queries for which the cached context will be used to serve and expected trends in the access rate) returns. For instance, cloud-based cache memory technologies such as Redis (https://redis.io) are stateful. Adding or removing an instance can be detrimental to the cost, performance efficiencies, and QoC, in addition to the post-scaling degradation [22], e.g., adding a 1GB cache instance to cache a 1KB of context information. Finally, adaptive context eviction involves determining what context to remove to maximize efficiency. In [21], we introduced ‘selective’ and ‘mandatory’ evictions for that matter. We would like to stress the feature ‘logical coherence of adaptive actions’ requirement of ACOCA [15]. Decisions at each stage must complement each other to maximize the overall efficiencies of the CMP discussed in this paper. Hence, our strategies in this paper are designed based on this requirement (extending the learnings from [21]) which is completely novel to this area.

### 1.3. Research Problems

Based on the facts above, caching is one of the functions that could be used to maximize both the cost and performance efficiencies of CMPs. However, the problem of achieving both of the objectives is nontrivial, i.e., PD→0 by caching all context but the cost of context management →∞. For instance, in [21], it is shown that the no-eviction policy was outperformed by the other benchmarks (e.g., least value first) under certain conditions. We also contend that caching any context, random or otherwise (e.g., based on popularity), will not yield the desired efficiencies due to several reasons based on Weerasinghe et al. [10], including (a) the differences between context caching to traditional data caching and (b) the cost of context management in the lifecycle, as opposed to selective caching, refreshing, or eviction alone. Therefore, the objective of this paper is to design, develop, and test an adaptive context-caching mechanism that:

- maximizes the cost efficiency (i.e., minimizes the cost of responding to context queries) of the CMP;
- maximizes the performance efficiency of the CMP, enabling it to respond in a timely manner to time-critical context queries;
- achieves a quasi-Pareto optimal state between the cost and performance efficiencies, and;
- minimizes the additional overhead of adaptation to the CMP.

For the sake of clarity, cost efficiency refers to minimizing all monetary costs incurred by the CMP when responding to context queries and managing context. As we will describe later in the paper, these costs involve retrieval costs, cache memory costs, processing costs, storage costs, penalties, etc. By performance efficiency, we refer to maximizing the quality of service (QoS) of the CMP when responding to context queries measured using the response latency, probability of delay (PD), hit rate (HR), etc.

We investigate and test a reactive adaptive context-caching algorithm in this paper. “Reactive” adaptation in the paper refers to performance metric-driven adaptations in response to observed variations in context (including that of the context-query load and context-query classes discussed later). ACOCA is a near-realtime algorithm that adopts the monitor, analyse, plan, execute, and knowledge (MAPE-K) process [23]. The contributions of this paper are as follows:

- develops mathematical models to compute costs for context caching and performance efficiently, cache-context adaptively;
- proposes a novel, scalable selective context-caching agent developed based on the TD3 algorithm in RL, different from adaptive data-caching approaches;
- proposes a cost and performance-efficient adaptive context-refreshing policy with refreshing-policy shifting;
- develops a time-aware context-management scheme to efficiently handle the context-management lifecycle costs;
- develops the ACOCA mechanism that encompasses the life cycle of context management. The mechanism is scalable and computationally less expensive compared to benchmarks that encounter the exploding cost of adaptive context management;
- verifies our theories and mathematical models using test results obtained from our experiments using a real-world-inspired synthetically generated large context-query load;
- compares the cost and performance efficiency of a CMP using ACOCA against several traditional data-caching policies and another RL-based context-aware data-caching policy for benchmarking.

The rest of the paper is structured as follows. Section 2 introduces the motivating scenario of this paper. Section 3 discusses the background of this work. Then, in Section 4, we lay the theoretical foundation for context refreshing and the policy-shifting algorithm, and in Section 5 we introduce the adaptive context-caching algorithm, mathematically formulating the solution. Section 6 describes the experimental setup and discusses the results obtained. We make references to our mathematical models in this section to validate them. Finally, we conclude with further directions in this area.

## 2. Motivating Scenario—Autonomous Car Parking

**Figure 3:**
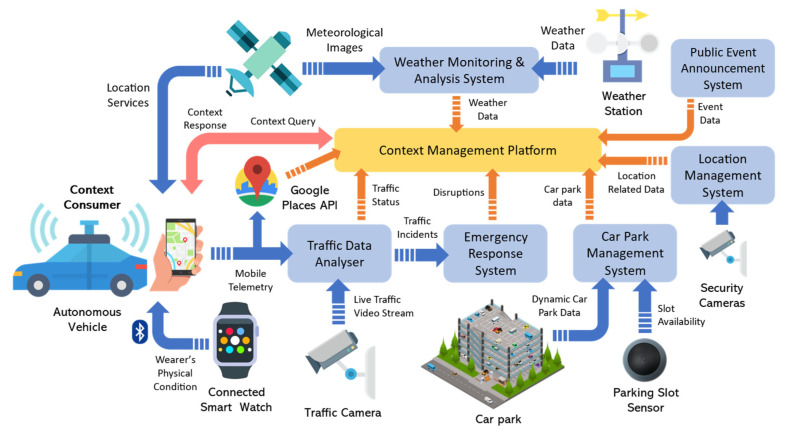
Context-aware searching for available car parking slots.

Let us consider the motivating scenario illustrated in Figure 3, which depicts a scenario during rush hours where drivers and/or autonomous vehicles send context queries to a CMP to find the “most suitable” car parking spots based on all the relevant context information. Similar to the scenario defined by Fizza et al. [24], this end-to-end autonomic IoT application may actuate gates to car parks upon arrival after routes are selected and updated based on the context information.

Context queries may be generated using a mobile IoT-based application or an onboard navigation system. Different context consumers can have various performance and/or quality of context requirements from the CMP, e.g., all context information should not be older than 20 s. Assume a driver intends to arrive at an event on time. The CMP is required to invoke relevant CPs to retrieve all available parking spots, features such as price, maximum parking hours of the parking facility, the driver’s physical condition, weather data, traffic conditions of the access roads, and any other known disruptions such as road closures. Each of these context attributes can influence the driver’s arrival time but also are dynamic enough to change frequently. For instance, recommending car parks for multiple autonomous vehicles and drivers in an area can also result in congestion at the entrance to the suggested car parks. Retrieving context data from all the relevant CPs to keep up with the dynamic environment for all the different drivers in a metropolitan area is expensive, considering the time and resource intensiveness of the process. Context caches provide a cost- and performance-efficient solution for both the CMP and the context-aware IoT-based applications.

The application scenario discussed here is nonintrusive. Hence, the cost, QoC, and QoS objectives defined in our research questions are of the utmost importance to achieve. For instance, the quality of the recommendations (e.g., the validity of vacant parking spots shown) made by the autonomous parking IoT application depends on the performance and accuracy of each stage of the ACOCA lifecycle once integrated into the CMP.

## 3. Related Work

Caching is a well-researched and developed strategy to optimize for demanding realtime loads. Given the interpreted nature of context information, the performance benefit of accessing context information from a cache memory over redirector mode would be in magnitudes [10]. Data popularity [3] and data request patterns [25] (which are indeed context with regard to data and queries) vary over time. Further to the complexity, sensed data originating from IoT devices (from which the context is derived) are also transient [11,26]. Previous work in adaptive data caching took data lifetime [11], properties of network queueing [17], popularity [3,4], and/or cost of caching [26] to make adaptive decisions. The problem however is that these parameters cannot be considered in isolation when managing context information compared to data caching. For example, caching non-transient popular items could maximize the hit rate (HR) by up to one. Transient context derived from multiple sources of data will always be HR<1 because cached context can be considered invalid due to several reasons. The most frequently investigated reason is invalidation (also referred to as ‘expiration’) [11,12]. It is the main criticism we find for probabilistically caching popular context entities in [27] where our opinion is supported by Wu et al. [21]. Scaling vertically (e.g., adding more CPUs to process context) or horizontally (e.g., indefinitely adding more cache memory) [28] is an attractive solution for maximizing performance efficiency but is a massively expensive option. Note that we also indicated the exploding cost of the adaptive context-management problem in Section 1.

### 3.1. Traditional Data Versus Context Information Caching

A significant body of research has been performed in the area of data caching and surveyed [29,30]. There exists a considerable number of promising techniques investigated in adaptive data caching, often referred to as context-aware data caching [4,15,19,30]. Interested readers are referred to our survey which compares and contrasts data caching with context caching and provides a concrete definition for adaptive context caching [15]. Table 1 indicates five significant contrasting features of data caching from context caching among many others to highlight why existing data caching techniques may not be fully applicable to caching context. Further, Weerasinghe et al. [10] categorically distinguish context caching from transient data caching (such as sensor data) and emphasise the unique challenges when caching context information. We discussed several more reasons in the previous section as well. For instance, eviction-based optimization (cache-all and evict strategy based on the write-one-read-many concept) can be both cost [31] and/or space inefficient [20] due to redundant cache-management operations.

Suggestive solutions can be found in the form of selective caching [32], from a library of data [5,33]. Value-based selection [5,34] and solutions developed using reinforcement learning techniques are well-studied [29] in each stage of the life cycle, however separately, using data. For instance, Somuyiwa et al. [35] selectively cache the least costly to cache, estimated as a function of the remaining lifetime. Kiani et al. [28] dynamically resize cache memory according to the query load. Zhu et al. [26], Sheng et al. [13], and Nasehzadeh et al. [36] provide evidence of cache-replacement strategies. There is a significant interest in leveraging machine learning (ML) techniques such as reinforcement learning (RL) using deep neural networks (DNN) to self-learn objective-oriented policies [30]. One major problem in implementing these strategies to cache context is the lack of data. Performance-metric-driven realtime algorithms such as adaptive caching rely on prior knowledge about the data and queries, e.g., transition probabilities among cache states as in [33]. Furthermore, context is dynamically interpreted information that cannot be attributed to concrete prior information when designing and implementing realtime solutions.

The intuitive solution to overcome the lack of prior knowledge is to continuously monitor. Caching policies that make cache-management decisions on each data item could be found in [11,12,37] but are very resource intensive having to monitor each piece of data. Individual context entities are monitored in the work of Khargharia et al. [27] where the probability to cache is estimated purely based on the popularity of context entity instances. It is infeasible to monitor each context information such as the context entities because

- there are billions of context entity instances (and growing) that are not scalable to monitor individually; hence, suitable indexing and binning techniques for context need to be investigated;
- unforeseen novel context information can be inferred with respect to retrieved data and context queries at any time;
- the same context information can be requested in context queries using different structures and formats (e.g., a context entity such as a vehicle can be defined using two or more different ontologies such as schema.org (https://schema.org/Vehicle, accessed on 27 February 2023) and MobiVoc (http://schema.mobivoc.org/#http://schema.org/Vehicle, accessed on 27 February 2023) by many consumers), which would duplicate monitoring;
- semantically similar context queries can request similar context information which, through monitoring, only previously observed context information discreetly using identifiers (e.g., as in [27]) will be incorrectly missed in the cache memory.

As a result, the overhead of adaptative context caching →∞ if the same technique is to be applied.

### 3.2. Overhead of Adaptive Context Caching

Another key problem concerning adaptive caching approaches could only be viewed after careful consideration. We highlight the critical problem of the computational complexity of RL-based adaptive data-caching approaches [21] due to large state and action spaces. The size of these models could explode if applied to context caching due to the novel and diverse context that can get inferred at any time. Further, authors in this area do not provide compelling evidence to prove whether the additional cost of cache adaptation using these approaches (i.e., especially in an exploding state and action space scenario) is justifiable against the earnings. We refer to the exponentially increasing total additional costs incurred by the CMP due to exploding models by size and the life cycle costs when managing context as the “exploding cost of adaptive context management” problem. Existing RL-based solutions to adaptive caching are not scalable to be directly applied to context caching [15] as a result. Statistical Agent (*StatAgn*) was proposed and tested in this light as it also elevates the problem of the long time to train [21]. The *StatAgn’s* strength to converge fast to a short-term optimal state is also its weakness to achieve long-term efficiencies when compared against the *ACAgn* and *DDPGAgn* [21,38]. We experimentally showed that the *StatAgn* is oblivious and incapable of self-learning to adapt to long-term patterns using experience. There is a compelling need to bridge the gap to develop a scalable, inexpensive, and yet lightweight enough solution when designing ACOCA, also justifying the additional cost of adaptation.

Based on the aforementioned, “scalability” in this paper is defined as the ability to execute the ACOCA algorithm on a significantly large number of heterogeneous context information without (a) stalling the CMP, and (b) incurring less than cost efficient costs of adaptation. Therefore, based on the definition of adaptive context caching in [15], “adaptation” is dynamically changing the context-selection model and context-refreshing algorithms in accordance with the features of the context-query load, context consumers, context providers, and context information (e.g., lifetime) in near realtime so that the cost efficiency goal(s) of the CMP measured by the quality of service (QoS) and quality of context (QoC) parameters are maximized.

### 3.3. Lack of Implemented Adaptive Context-Caching Mechanisms

The rationales discussed previously in this section form the basis for why adaptive context caching has not been implemented in CMPs and is only sparingly investigated in the literature despite its potential. Weerasinghe et al. [10,15] provide evidence of further complexities in managing context in the cache. In addition, architecture and design concepts contribute significantly to the lack of knowledge in ACOCA. CMPs such as FIWARE Orion [9] and Nexus [8] execute context queries entirely in database mode [39]. Data required for deriving context information are readily available in the context storages and are polyglot persistent, specializing in different domains (e.g., generic enablers in FIWARE Orion). Further, FIWARE Orion adopts a building-block approach [40] to develop specific instances of CMSs. It is therefore evident that the developers were motivated by optimizing the CMPs based on technology diversification based on factors such as type of data and domain. A specialized context-storage approach still does not solve the overhead of accessing an enlarging IoT data storage due to continuously streaming data. Hence, the scalability of accessing the context databases through a single point of access (i.e., an interface) is limited.

In summary, this paper identifies and addresses a significant research gap, which is the lack of a comprehensive solution to adaptively cache context. We explained why ACOCA is a unique problem compared to adaptive (i.e., context-aware) data caching. This work aims to develop and test a cost-efficient adaptive context-caching strategy that maximizes the performance efficiency of the CMP when responding to context queries of the context consumer. The cost efficiency in ACOCA encompasses the requirement of breaking-even the additional cost against the earnings generated as a direct result of ACOCA.

In the next sections, we will introduce the ACOCA algorithm. First, we introduce the context-refreshing policies in Section 4 in order to develop and present several theories which will be used later in Section 5.

## 4. Adaptive Context Refreshing with Policy Shifting

Adaptive context is refreshing with policy shifting could be broken down into two adaptations: (a) adaptive refresh-rate setting and (b) refresh policy shifting. Refresh policy shifting involves shifting between proactive refreshing with shift [12] and reactive refreshing [11,12] policies to maximize the cost efficiency of the refreshing process based on several parameters. The adaptive refresh-rate setting is applicable only when the proactive refreshing with shift policy is applied in the cached context.

For the consistency of figures in this section, the following coloured arrows are used to represent the following. A dark blue solid arrow is used to represent requests for context from the consumers with SLA_1_ and pink for requests for context from the consumers with SLA_2_. Orange solid arrows represent context retrievals and dashed orange arrows represent planned (not executed) retrievals. Grey solid arrows denote the arrival of the context at the CMP from the CP whereas yellow solid arrows represent context retrievals from alternate CPs other than that from which the cached context is inferred from. Purple arrows with a round head denote sampling events of a CP. The solid diagonal green line represents the loss of freshness of a context.

Table 2 summarizes all the notations used in this paper for our discussion.

### 4.1. Why Adaptive Refreshing with Policy Shifting?

We develop a modified proactive refreshing with a shift policy in this work based on several reasons.

First, in our previous work [11], we discovered that the full-coverage refreshing policy which is adaptive to the dynamic lifetime of a context was the most performance-efficient among reactive and nonadaptive (redirector) approaches.

Second, Medvedev et al. [12] investigated proactive refreshing with shift for raw context cache, where it was discovered that the proactive refreshing with shift was more cost efficient than the full-coverage policy. The work in [11,12] is, however, incomparable due to two reasons: (a) our previous work [11] evaluated the policies with dynamically varying context lifetimes, for which the refresh rate adapts and significantly increases the validity (i.e., measured by reduced invalidity rate) of cached context; comparatively, [12] assumes a static context lifetime during a planning period (PP) and (b) evaluation of proactive refreshing with shift has only been performed for a configured value of $t_{g}$, that is, the gap time between the time a context is expired and refreshed; there is no evidence about the sensitivity of the solution to increasing and decreasing the value of $t_{g}$ or a rationale provided on how to select a suitable value for $t_{g}$.

Based on the results obtained for the reactive refreshing policy in our previous work [11], it was evident that the gap time $t_{g}$ is subject to the request rate (λ) and the applied freshness threshold ($f_{thr}$) set by the context-requesting context consumer who individually defines the maximum tolerated age of a context. We further learned in the proof-of-concept (https://bit.ly/2ZgaOmt) developed for adaptive context caching (ACOCA) [11], that (a) access rate (AR)—the rate of accessing a particular context and (b) $f_{thr}$ are the parameters that determine $t_{g}$. In the presence of heterogeneous context consumers with different tolerances to the age of the context (depending on factors such as the time criticality of the context query), estimating $t_{g}$ with even two of these parameters is not trivial, as also found in [12].

Given the drawbacks of previous investigations such as the lack of practical implementation [18], computational complexity [11], and challenges in developing the proactive refreshing with shift policy [12], we investigate a simple, yet innovative, way to overcome these issues and challenges. The solution is twofold, as described below.

### 4.2. Adaptive Context Refresh Rate Setting

First, we calculated the expected $f_{thr}$ (E[$f_{thr}$])—the minimum accepted relative freshness (based on lifetime) by the context consumers for each context. The context-query execution monitor (CQEM) profiles the context (as we will show in Section 6). It also profiles the quality requirements that are applied when the context is retrieved. CQEM aggregates this data into a probability distribution of each quality parameter within the PP. The E[$f_{thr}$] of a context *i* considering all the different SLAs ($n\;\in \left\{1,2,\ldots ,N\right\}$) can therefore be derived as follows in (1) where $P\left(f_{thr,n}\right)$ is the probability of *f_thr_* from the n^th^ SLA being applied to an accessed context:

(1) $$E{\left[f_{thr}\right]}_{i}=\sum P\left(f_{thr,n}\right)\times f_{thr,n}=\frac{\sum f_{thr,n}\times AR_{n}}{\sum AR_{n}}$$

**Figure 4:**
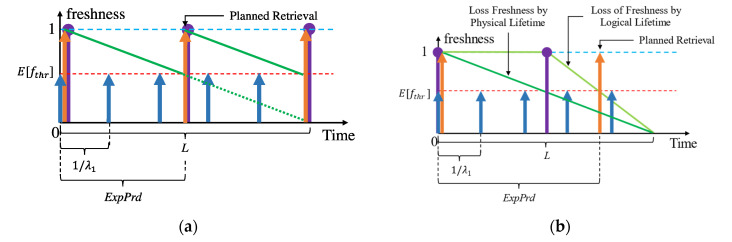
Generic examples for (**a**) physical lifetime and (**b**) logical lifetime when the CP samples periodically and periodically, respectively.

As we illustrate in Figure 4, the planned retrievals for refreshing will occur when the expiry period (*ExpPrd*) [11,12] is calculated using $E{\left[f_{thr}\right]}_{i}$ elapses. It depicts the loss of context freshness against time when the CP samples (a) aperiodically and (b) periodically (assuming the logical lifetime was estimated accurately to the physical lifetime). The *ExpPrd* of a context with respect to an SLA or a collection of SLAs (as in the case with $E{\left[f_{thr}\right]}_{i}$) is the time during which a context is considered “fresh enough” (referring to the subjectiveness to the context consumer) for responding to a context query. Consider the following example: for N = {1, 2, 3, 4}, the access rates (ARs)—the rate at which a piece of context information is requested from the CMP per second—are 0.8, 1.2, 0.4, and 3.0, respectively, for context *i*, whereas $f_{thr,1}=0.5$, $f_{thr,2}=0.6$, $f_{thr,3}=0.8$, and $f_{thr,4}=0.7$. Therefore, $E\left[f_{thr}\right]$ is:

$$E\left[f_{thr}\right]=\frac{\left(0.5\times 0.8\right)+\left(0.6\times 1.2\right)+\left(0.8\times 0.4\right)+\left(0.7\times 3.0\right)}{\begin{array}{c} \left(0.8+1.2+0.4+3.0\right)\\ =\frac{0.4+0.72+0.32+2.1}{5.4}=0.65 \end{array}}$$

Assuming the lifetime of the context is 10 s and *age* = 0, then the $ExpPrd=lifetime\times \left(1-E{\left[f_{thr}\right]}_{i}\right)=10\times \left(1-0.65\right)=3.5\;s$. The planned retrieval using the proactive with shift policy would occur after 3.5 s from the last context retrieval as a result if the context-provider samples its environment on demand for requests (later referred to as aperiodic sampling).

#### 4.2.1. Handling the Different Context Lifetimes

There are two ways to view the lifetime of a context value: (a) physically and (b) logically. Physical lifetime is the actual time that a context value takes to change its value. Physical lifetime is commonly used (e.g., as in [12]) and estimated (e.g., as in [11]) because it is (a) easy to understand and apply and (b) synchronous with the real-world environment. However, physical lifetime is most accurately estimated only when CPs perform aperiodic sampling of their environment. Logical lifetime is the perceived lifetime of a context by the CMP which is typically not equal to the physical lifetime. This phenomenon occurs when the CP samples the environment or data from the CP is ingested periodically. For example, consider a quick-stop parking spot that is occupied every 90 s, but the sensor samples only 60 s. The perceived lifetime would be between 60–90 s based on the techniques used in [11]. Given there is no other way to retrieve an updated sample value before 60 s from the CP, the logical confidence in the value is 100%. Any estimated lifetime within $\left[60,\;90\right]$ is therefore subject to confidence. Therefore, $f_{thr}$ is a measure of this confidence in the last retrieved/derived context. Note that the physical and the logical lifetime of a context value is equal only when the context is retrieved or derived aperiodically. We consider the logical lifetime in our discussions since, (a) the CMP as a caching agent (i.e., a middleware) only has the perceived view of the context lifetime, and (b) it is useful in minimizing the number of refreshing retrievals, e.g., as depicted in Figure 4 where the *ExpPrd* of (b) is greater than (a).

#### 4.2.2. Synchronizing Context Refreshing to Maximize QoC and Using Alternate Context Retrievals

**Figure 5:**
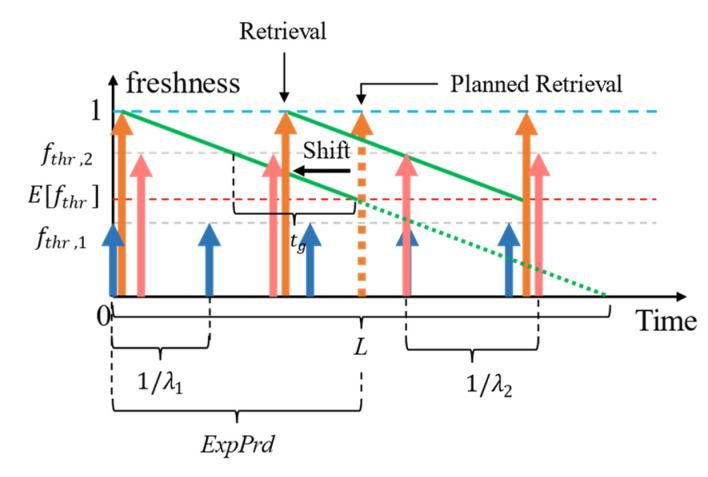
Modified proactive refreshing with shift using $E\left[f_{thr}\right]$.

When the variance of the distribution of $f_{thr}$, $\sigma^{2}>0$, several $f_{thr}$ values applied to the context can be greater than the E[$f_{thr}$]. Requests for context with $f_{thr}$ < E[$f_{thr}$] can always be served using the context cache. We refer to the consumer SLAs having lower tolerance to the age of the context compared to the expected value ($f_{thr}>E\left[f_{thr}\right]$) as being more expensive than the average SLAs. The number of expensive SLAs ($N_{exp}$) is always $N_{exp}>0$ unless the $f_{thr}$ of all applicable SLAs are homogeneous. The difference between the $f_{thr}$ of expensive SLAs against the $E\left[f_{thr}\right]$ creates the gap $t_{g}$, during which time we can expect cache misses that trigger retrieval and shift (Figure 5).

The problem now is how to set $t_{g}$? We mentioned that $t_{g}\;$depends on the AR and the $E\left[f_{thr}\right]$—both of which are subject to the features of the context-query load (e.g., the composition of different context consumers making context queries and criticality levels of context queries—the relative importance of the context information to the context consumer to make a decision, etc.). Therefore, the HR of a context during the $t_{g}$ of two different $f_{thr}$ values can be given based on [41]:

(2) $$HR\left(t_{g}\right)=e^{-AR\times t_{g}}$$

As proof of the above claims, let us consider the above example again. The hit rate (HR) of the context when *n* = 1 or *n* = 2 is always 1.0 (i.e., 100%). In a proactive refreshing with no reuse situation [12] (which is less cost efficient than proactive refreshing with shifting), the HRs when *n* = 3 and *n* = 4 during the respective $t_{g}$s would be, $HR_{i,n=3}=e^{-0.4\times 0.8}=0.72$ and $HR_{i,n=4}=e^{-0.7\times 3.0}=0.12$. When retrieval is shifted upon a cache miss, then the $HR_{i,n=3,4}\to 1$ during $t_{g\;}$because:

$$t_{i,\;n=3,4\;}=\left[L\times \left(1-0.7\right)\right]-\left[L\times \left(1-0.8\right)\right]=0.1L\;HR_{i,n=3,4}=e^{-0.4\times 0.1L}=e^{-0.04L}$$

where *L* is the lifetime of the context and $t_{i,n=3,4}$ is the gap between the expiry periods of *n* = 3 and *n* = 4 for *i*. Since the lifetime of a context is transient and small, considering L→0, then the overall HR during this gap is $HR_{i,n=3,4}\to 1$.

**Figure 6:**
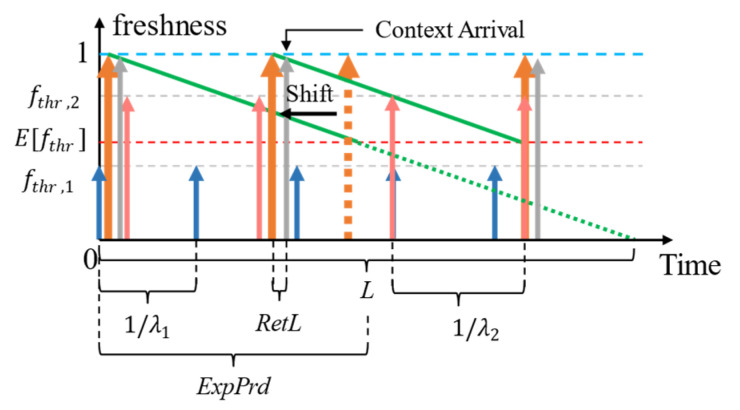
Proactive refreshing with shift policy when the CP samples only in response to a context request.

**Figure 7:**
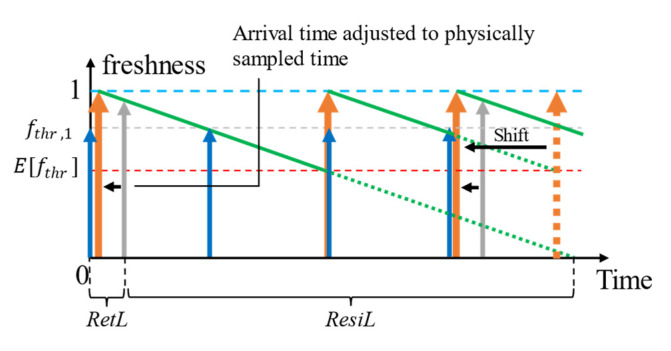
Modified proactive refreshing with shift policy adjusted for age loss during context retrieval.

In the work of Medvedev et al. [12], it is assumed that the context provider responds with the perfectly fresh context data (*age* = 0). This refreshing policy, where it is assumed the context provider samples the environment in response to a request for data, is indicated in Figure 6. This approach, however, is not entirely practical due to two reasons: (a) network latency during which time the context accumulates age and (b) the difference between the sensor-sampled time and the retrieval-requested time when the context provider senses the environment only periodically. Figure 7 indicates the policy adjusted for when the *age* = retrieval latency.

**Figure 8:**
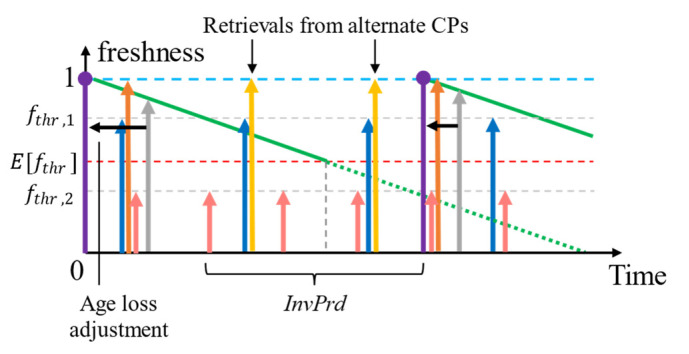
Modified proactive refreshing with shift policy with alternate CP retrieval when the *ExpPrd* < *SI*.

Resolving this issue with periodically sampling context providers is nontrivial. For example, the sampling interval (SI) of a sensor and the lifetime (*L*) of the property which it senses can be different where *SI* = 30 s and L = 60 s. When $E\left[f_{thr}\right]=0.65$, the refreshing could occur every $60\times \left(1-0.65\right)=$ 21 s. Assuming the retrieval occurred at *time* = 0, the retrieved value of the first refresh operation is at time = 21 s, which is 21 s old (because the sensor senses only when *time* = 30 s). We refer to this gap of time as the invalid gap for context provider (CP), where it cannot be used for refreshing. CPs are listed in order [6] since CoaaS need to be cost and quality-aware when selecting CPs for context retrieval. We use this to retrieve the context from the next best CP, the context from which could also be in the cache. This process is illustrated in Figure 8. Depending on whether the data from the alternate CP is already cached and selected for caching after retrieval or otherwise and our modified policy would temporarily operate either as proactive refreshing with reuse or proactive refreshing without the reuse policies investigated in [12]. Although this implicit policy shift is very short-lived, it is still a part of the adaptive-refreshing policy shift adopted in our work for the best cost (since when HR→1, need to retrieve decreases reducing the total cost of retrievals), quality (we avoid responding with any stale context), and retrieval efficiency.

**Figure 9:**
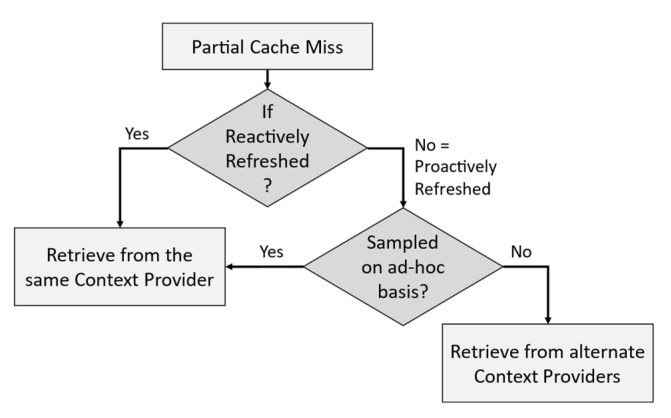
Decision tree of resolving the CP for refreshing in the event of a partial miss.

Figure 9 below depicts the decision tree for resolving the CP to retrieve among the ordered list of CPs by quality and cost (depicted in Figure 8) in response to partial cache misses. We calculate the *ExpPrd* for a context from the time of its actual origin (e.g., for a low-level context such as a temperature measurement, *lifetime* = 0 when it was measured). When *ExpPrd* < *SI*, the logical lifetime is adjusted to the physical lifetime to maximize the QoC and minimize the refresh rate such that the reactive refreshing can always retrieve from the same CP irrespective of the sampling technique. A full-cache miss will also lead to retrieval from the same CP looked up in the cache.

#### 4.2.3. The Problem of Alternate Context Retrievals

Retrieval/ingestion of context data from CPs can take two forms: (a) periodically or aperiodically fetched from the CP or (b) subscribing to a context data stream generated by the CP (e.g., using MQTT). Context data streams are always synchronous, whereas fetching can be both synchronous and asynchronous depending on the refreshing policy. Reactive refreshing is asynchronous since the refresh operation is triggered at different intervals depending on the $f_{thr}$ demand that caused the cache miss. Proactive refreshing synchronously retrieves from a CP with respect to the context lifetime, as long as it is unchanged.

We discussed that additional retrieval operations from alternate CPs can occur during invalid periods (*InvPrd*) [11] when the proactive refreshing policy is executed. The *InvPrd* is defined as the time until the subsequent retrieval from the point the freshness threshold is no longer met. In a multi-SLA scenario, the *InvPrd* starts when at least one of the $f_{thr}$ are not met (the earliest to meet the $f_{thr}$ is the most expensive SLA). Additional retrievals during this time are an additional cost that is not cost efficient. Let us extend our example above and assume that all the alternate CPs cost the same as the first selected. The HR of a context in cache given an SLA can be derived as follows in (3) where *AcsInt* is the average time between two requests for the same context and *RetL* refers to the context-retrieval latency. Equation (3) holds only when *ExpPrd* ≥ *AcsInt*, or else HR = 0.

(3) $$HR=\frac{AcsInt}{ExpPrd}=\frac{1/AR}{\left[\left(1-f_{thr}\right)\times L\right]-RetL-age}$$

Note that in (2), we showed the HR during the gap time between two $f_{thr}$, which can be used to calculate the HR subsequent to the *ExpPrd* in concern.

Based on (3), the total cost of retrievals per unit time (i.e., second) during an *ExpPrd* can be derived as follows, where $Cost_{ret}$ is the cost of context retrieval:

(4) $$Cost=AR\times \left(1-HR\right)\times Cost_{ret}$$

Assuming RetL, age→0, and considering the cost of retrieval $Cost_{ret}=\$0.5/request$, the total cost of retrieval for each SLA per second are AUD 0.38, AUD 0.58, AUD 0.16, and AUD 1.48, respectively, the most expensive when *n* = 4.

Comparatively, refresh operations using the proactive policy are most likely to retrieve reactively from alternate CPs during the 9 s invalid period. For each SLA however, the IRs are 0 s, 6 s, 18 s, and 12 s. They amount to AUD 0, AUD 0.12, AUD 0.12, and AUD 0.60 in additional retrieval costs per second. The accumulated cost is AUD 0.84.

**Figure 10:**
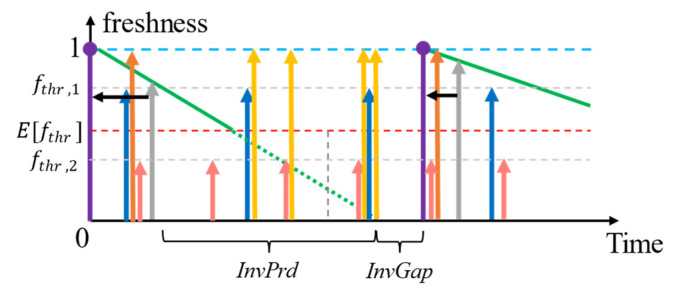
Attempting to refresh proactively with alternate CP retrievals when L < SI.

We illustrate an example where the L < SI in Figure 10. Consider L = 25 s, and therefore, in addition to the IR created as a result of $f_{thr}$, which are 12.5 s, 15 s, 20 s, and 17.5 s, there is an invalid gap, *InvGap* = 5 s for all the SLAs. The additional retrieval costs are AUD 0.47, AUD 0.8, AUD 0.33, and AUD 2.25, totalling to an additional retrieval cost of AUD 3.85. This additional cost is greater than the total cost of retrieval using reactive refreshing, even if each SLA is to be applied independently.

Here, *InvPrd* is the time until there is no longer any freshness left in the context (end of the lifetime) from the point where the most expensive SLA met with $f_{thr}$. The L < SI scenario creates an invalid gap (*InvGap*), during which the HR = 0 for any access to context *i* in the cache. So, it is inevitable to retrieve from at least an alternative CP.

We showed that optimizing the refresh rate for a variable lifetime and distribution of QoC parameters can lead to cost inefficiencies when alternate context retrievals are involved. Yet, alternative retrievals are inevitable in this technique to maximize the QoC of cached context information. So, we will investigate an improvement to our policy in the next subsection.

### 4.3. Adaptive Refresh Policy Shifting

We have indicated the expected HR during $t_{g}$—the gap between reaching two $f_{thr}$s is $e^{-\lambda t_{g}}$ in a multi-SLA cache retrieval scenario. Given the miss rate, MR = 1–HR, the expected MR during a $t_{g}$ using (2) is,

(5) $$MR=1-e^{-\lambda t_{g}}$$

We considered Poisson processes for λ and hence, AR would follow a Poisson distribution. When there are more than two SLAs that are applicable for retrieval and refreshing a context, the resultant process is also a Poisson process, the intensity of which $\lambda =\sum \lambda_{n}$, where *n* is the index of an applicable SLA. Therefore, when the times at which the $f_{thr}$ are met in descending order of the expensiveness of the SLAs, it adds to the current expected MR during $t_{g}$. Consider that MR_1_ is the miss rate expected during the $t_{g}=t_{1}$ the two most expensive SLAs; then, $MR_{t_{1}}=1-e^{-\lambda_{1}t_{1}}$ where $\lambda_{1}$ is the AR using the most expensive SLA (*n* = 1) because of the probability of a cache miss during $t_{1}$ is a result of context requests using the most expensive SLA. During the gap between the second-most-expensive and the third-most-expensive SLAs ($t_{2}$), the probability of a cache miss is a result of all the context requests using SLA_1_ and SLA_2_. According to [42], the result request rate of two superimposed Poisson processes (i.e., the context-requests request rates using SLA_1_ and SLA_2_) is their addition. So, the request rate expected to cause cache misses is $\lambda_{1}+\lambda_{2}$ (also as per $\lambda =\sum \lambda_{n}$). Then, the MR during $t_{2}$ is $MR_{t_{2}}=1-e^{-(\lambda_{1}+\lambda_{2})t_{2}}$. Based on the above, and the fact that $t_{1}$ is followed by $t_{2}$, MR as a function of time during the time $t_{1}+t_{2}$ can be given as follows:

(6) $$MR=\left(1-e^{-\lambda_{1}t_{1}}\right)+\left(1-e^{-(\lambda_{1}+\lambda_{2})t_{2}}\right)$$

where $\lambda_{2}$ is the AR using the second-most-expensive SLA (*n* = 2). Accordingly, considering *N* number of SLAs, the MR by the time the $f_{thr}$ of the cheapest SLA is met can be defined as follows:

(7) $$MR=N-\overset{N}{\underset{n=1}{\sum }}e^{-\left(\overset{n}{\underset{m=1}{\sum }}\lambda_{m}\right)\times t_{n}}$$

The total cost of retrieval during any $t_{g}$ is:

(8) $$TotalCost_{ret}=MR\times \left(\overset{n}{\underset{m=1}{\sum }}\lambda_{m}\right)\times t_{n}\times Cost_{ret}$$

The N number of SLAs results in, at most, N-1 $t_{g}s$ when L>SI or N number of $t_{g}s$ when L≤SI. Considering the L>SI scenario, then the additional cost of retrieval would be caused by K number of gaps, where K<N-1 and the number of SLAs having $f_{thr}>E\left[f_{thr}\right]$. Then the total additional cost of retrieval until the automatic retrieval is:

(9) $$AddTotalCost_{ret}=\overset{K}{\underset{k=1}{\sum }}TotalCost_{ret}$$

**Figure 11:**
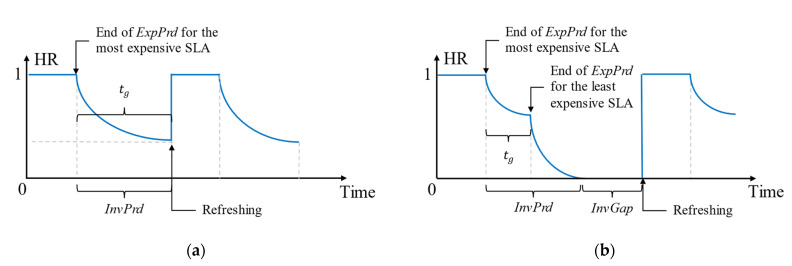
The variation of HR for a 2-SLA scenario where, (**a**) SI < L, and (**b**) SI ≥ L.

Considering (2) and (5)–(9), the two scenarios discussed in Figure 7 and Figure 9 can be indicated using the probability for a cache hit (also the HR), as follows in Figure 11 considering two SLAs for simplicity.

According to Figure 11, it can be proven that the proactive refreshing with shift would operate at the same cost efficiency when L ≤ SI or $E\left[ExpPrd\right]\leq SI$.

**Figure 12:**
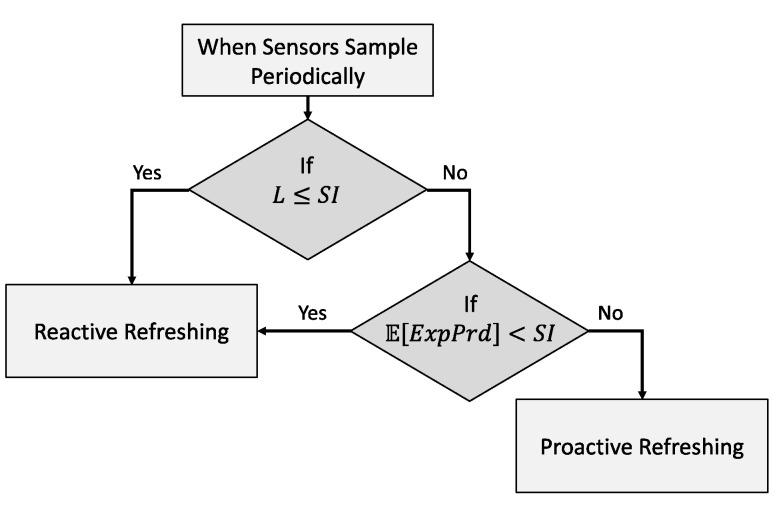
Decision tree for resolving the refreshing policy when it depends on the *ExpPrd*.

We can summarize the conditions that decide the context-refreshing policy as follows in Table 3. Figure 12 further depicts the decision tree by which the refreshing policy is resolved for those stated as “Depends on *ExpPrd*” in the table.

As indicated in the decision tree below in Figure 12, the *ExpPrd* for E[$f_{thr}$] is calculated when evaluating the policy. It was obvious by our example, and the formulas above, that proactively refreshing cached context when the L ≤ SI is cost inefficient. The reactive policy is, comparatively, cost efficient or equal to the proactive refreshing policy as it delays the retrieval interval further than SI until a cache miss occurs.

When L > SI, we compare SI with the expected residual lifetime, E[*ExpPrd*]. E[*ExpPrd*] is calculated as follows:

(10) $$E\left[ExpPrd\right]=\left(L-E\left[RetL\right]\right)\times \left(1-E\left[f_{thr}\right]\right)$$

(11) $$E\left[RetL\right]=\frac{\sum_{r=0}^{R}Ret_{r}}{S};where\;S\leq R$$

(12) $$Reliability=\frac{S}{R}$$

where S is the number of successful retrievals and R is the total number of context retrievals attempted.

Context providers have a degree of reliability—the probability of responding to a context data request on the first attempt. CoaaS is implemented to retry fetching from the selected CP, up to twenty attempts in a single retrieval operation. Therefore, the total *RetL* of a retrieval operation is defined as follows:

(13) $$RetL=\sum_{x=0}^{y}ResponseTime_{x};where\;0\leq y<20$$

For example, when the CP is unresponsive due to some reason (e.g., hardware malfunction or network failure), the reliability of the CP would be zero (0/R), whereas the E[*RetL*]→∞ (when $\sum_{r=0}^{R}RetL_{r}/\underset{S\to 0}{\lim}S$).

**Figure 13:**
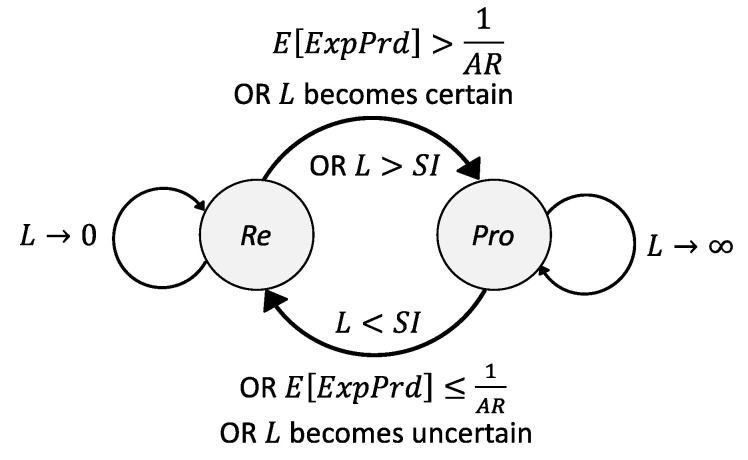
Transitions diagram between the refreshing policies when shifting in response to different conditions of the context.

The calculated E[*ExpPrd*] provides a probabilistic value of the useful lifetime of a context in the cache memory which, by the rationales above, dictates the cost and retrieval efficiency of the policies. It is hence the reason that the proactive refreshing with shifting is employed only when E[*ExpPrd*] ≥ SI, as it guarantees there will not exist any invalid periods [11] (referring to those caused by uncertainty in an inferred lifetime [43]), during which time the policy incurs additional costs. Figure 13 below indicates the transition diagram illustrates the transitions between the two refreshing policies in response to different conditions that the context information goes through.

The certainty of L refers to the confidence in the inferred lifetime (confL) which we described in [43]. When $\sigma_{L}\to 0$, confL→1 which maximizes the performance efficiency benefit and the invalid rate (IR) →0. L can largely vary between the L>SI and L<SI conditions such that the cost and performance efficiency achieved from the proactive refreshing with shift policy diminish worse than the reactive policy alternatively, while being IR >0 when confL→0. Therefore, in order to achieve more accuracy in the inferred lifetime [11] and minimize the IR, it is more rational to use the reactive refreshing policy when confL→0, as indicated below in Figure 13.

In the next section, we present the ACOCA algorithm which encompasses this adaptive context-refreshing policy.

## 5. Reactive Context Cache Selection

In this section, we introduce the reactive context-selection algorithm for caching.

There are two approaches to addressing this problem: (a) selecting a subset of context information from a candidate set to cache (limited caching actions), or (b) separately evaluating each context information to determine whether it would positively contribute to the cost efficiency of the CMP if cached (unlimited caching actions). The former is computationally expensive since each combination of context information from a candidate set of contexts needs to be tested to maximize the resultant $\bar{Gain}$. The process can be at least NP-Hard (because for a set of I candidate contexts values at a given time, there are $C_{r}=I!/r!\left(I-r\right)!$ combinations to evaluate for caching, where r is the number of context values potent to cache). The latter approach is computationally less expensive. We opt to individually evaluate context information as it is accessed to respond to context queries. Based on our objectives defined in Section 1, the key challenge in reactive context selection for caching is how to filter context that will be cost and performance inefficient to manage from being cached.

### 5.1. Confidence to Cache

We develop a value-based approach to estimate the confidence to cache a certain context *i* denoted by $conf\left(i\right)$ in the context cache. The decision is, therefore, binary, as indicated in (16). The $conf\left(i\right)$ is derived as a linear function based on the concepts from the previous work which defined the need to refresh (NRT) [12], and the value of a cached item [37]. However, our approach principally learns a time-variant hyperplane in the five-dimensional decision space to maximize the separation between contexts that would yield cost and performance efficiencies for the CMP and those that would not.

The $conf\left(i\right)$ is defined as follows in (14), where CE is the cache efficiency, RE is the retrieval efficiency, AT is the access trend (i.e., the trend of the time series of the access rate), Unreli is the unreliability of context retrieval, and Cmpx is the average complexity of the context queries for which the context would be used to respond with. We will explain them further in this paper. The derivation of this formula is provided below using (17)–(20).

(14) $$conf\left(i\right)=\mu E\left[CE\right]+\omega E\left[RE\right]+\kappa E\left[AT\right]+\delta E\left[Unreli\right]+\rho E\left[Cmpx\right]$$

where κ,μ,ω,ρ,δ are weights assigned to each of the parameters in the equation and,

$$conf\left(i\right),\;AT,CE,Cmpx\;\in R_{0}^{+}\;0\leq \kappa ,\mu ,\omega ,\rho ,\delta <1\;0\leq Unreli\leq 1$$

As indicated earlier, the caching or not caching is decided based on a threshold that is learned partly (i.e., θ–the cache distribution bias) in the same process as the weights. The threshold (ϑ) is a function of θ and the $conf\left(i\right)$ distribution.

**Figure 14:**
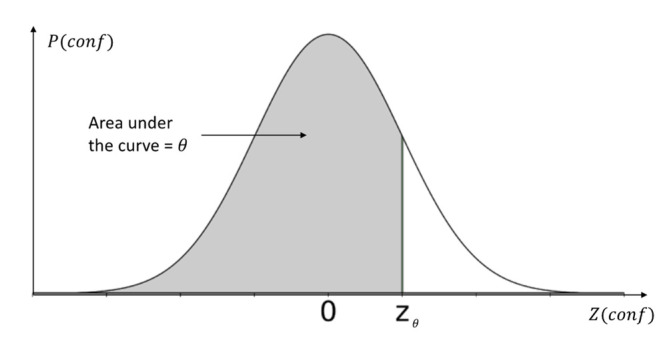
Calculating ϑ using the estimated θ.

The binary selective context-caching decision is:

(15) $$cache\left(i\right)=\left\{\begin{array}{c} true\;;conf\left(i\right)>\vartheta \;\;\\ false\;;otherwise\; \end{array}\right.$$

where *N* is the sample size. Given 0≤θ≤1 as the rest of the weights where P(*conf*) is the probability distribution of *conf* values in the sample and θ is an estimated probability (i.e., the area under the z-curve). Therefore, ϑ is solved by deriving the *conf* value for θ in the z-distribution. Figure 14 illustrates an example of this calculation and is further explained in (16) where $\sigma_{conf}$ is the standard deviation of the sample of *conf* values and $Z_{\theta }$ is the calculated z-value for the estimated θ.

(16) $$\vartheta =\bar{Conf}+\sigma_{conf}\times Z_{\theta }$$

For example, if θ=0.33, $Z_{\theta }=-0.44$. Assuming $\bar{Conf}=0.8$ when $\sigma_{conf}=0.5$, then the cache decision threshold $\vartheta =0.8+\left(0.5\times -0.44\right)=0.58$.

#### 5.1.1. Deriving Confidence to Cache

Our objective is to select and cache the context information that are most potent to improve the cost efficiency, performance efficiency, and overall quality of context. Since any context information that maximizes a set of parameters can be selected (unconstrained selection) for caching, we first need to identify the set of objectives that suitable candidate context information should maximize.

First, in previous work in value-based approaches, such as the least value first (LVF) [37], NRT [12], and the value-based replacement in [34], the value functions were defined linearly giving empirical evidence. Second, in [38], we explained how the value of a context in the cache can be low when (a) the context is relatively unpopular to generate adequate earnings with respect to the cost of managing it in the CMP, (b) the remaining cache lifetime→0 or has elapsed, and/or (c) the context information can be retrieved/derived fast enough (depending on the performance requirements). Later in Section 5.3 and Section 5.4, we show that CE and RE are derived based on this concept. CE and RE are mutually exclusive variables that can impact achieving the efficiency goals equally. For example, a very cache-efficient context can be retrieval inefficient which could lead to significant overheads, both cost and performance wise to the CMP. On the other hand, a retrieval-efficient context may not be cache efficient, leading to worse than redirector-mode performance efficiency for the CMP. Therefore, the objectives of maximizing RE and CE can be indicated as follows,

(17) $$\underset{I}{\max}\;CE_{i},\;\underset{I}{\max}\;RE_{i}$$

The exact impact of the parameters on achieving our objectives is subjective to the context of the context-query load. Therefore, by scalarizing the objectives by assigning weights, the basic form of (14) can be indicated as below. We refer to the product of the scalarized objectives as the *confidence to cache* ($conf\left(i\right)$).

(18) $$conf\left(i\right)=\mu E\left[CE\right]+\omega E\left[RE\right]+error$$

Second, related work, especially those that perform value estimation in reinforcement learning, uses random values to learn the error by exploration, e.g., [44]. Given the time-variant nature of context-query loads, we opt to estimate this error using imperative knowledge to (a) minimize the time to converge, (b) eliminate the complexity of estimating an unbound error value (error∈R) using an approach such as in [44], (c) minimize the estimation errors when attempting to converge to an unbound optimal value, and (d) minimize the inefficiencies to the CMP during the learning period.

From our previous work [45], we identified that the impact of caching on efficiency correlates positively with the complexity of the context query (*Cmpx*). Therefore, a piece of context information that is potentially used to respond to more complex context queries (*Cmpx(i)*) is potent for caching. Then, [21,38] define events that result in a sudden “spike” in popularity during a very short period of time (considering an accident as an example). As AT correlates with the hit rate, it is also a potent indicator for such a scenario [27]. Finally, the reliability to derive the context information in time (*Reli)* is subject to the time and the context itself. For example, Reli→0 when it is the rush hour (time dependent), or in response to a situation such as an accident (context). In both cases, networks can get congested resulting in packet loss and delays. When the reliability decreases (unreliability Unreli=1−Reli, increases), it is potent to cache the context to minimize retries, network utility, and delay associated with it.

(19) $$\underset{I}{\max}\;AT_{i},\;\underset{I}{\max}\;Unreli_{i},\;\underset{I}{\max}\;Cmpx\left(i\right)$$

All the additional objectives of context selection in (19) attempt to select a context that maximizes the parameters, *AT*, *Unreli*, and *Cmpx*, similar to *CE* and *RE* in (17). Therefore, we can modify the scalarized objective in (18) as follows in (20).

(20) $$conf\left(i\right)=\mu E\left[CE_{i}\right]+\omega E\left[RE_{i}\right]+\kappa E\left[AT_{i}\right]+\delta E\left[Unreli_{i}\right]+\rho E\left[Cmpx\left(i\right)\right]+err$$

We assumed that err→0 to arrive at (14) using this formula. Therefore, considering $v\left(i\right)$ is the feature vector of a candidate context *i* to cache where,

(21) $$v\left(i\right)=\left\{AT_{i},\;CE_{i},\;RE_{i},\;Unreli_{i},\;Cmpx\left(i\right)\right\}$$

Considering φ is the set of the values of all the learned weights and $conf\left(i\right)$ is the dot product of a given φ and $v\left(i\right)$. Considering the sample size from the context-cache decision history is *N*, (23) indicates the problem that we solve in our solution.

(22) $$v\left(i\right)⊙\varphi =conf\left(i\right)$$

(23) $$\left[\begin{array}{ccc} AT_{1} & \begin{array}{ccc} CE_{1} & RE_{1} & Unreli_{1} \end{array} & Cmpx\left(1\right)\\ \vdots & \vdots & \vdots \\ AT_{n} & \begin{array}{ccc} CE_{n} & RE_{n} & Unreli_{n} \end{array} & Cmpx\left(n\right) \end{array}\right]⊙\left[\begin{array}{c} \begin{array}{c} \begin{array}{c} \kappa \\ \mu \end{array}\\ \omega \end{array}\\ \delta \\ \rho \end{array}\right]=\left[\begin{array}{c} conf_{1}\\ \vdots \\ conf_{n} \end{array}\right]$$

Each vector $v\left(i\right)$ is calculated using the observed data and the resultant set of cache confidence values are derived for a nonconverged version of φ. The problem is to solve the optimum set of weights and θ that maximize the average gain from responding to context queries. We call this the φ+θ problem (shorthand for $\varphi +\left\{\theta \right\}$, which is the set of all the learned weights and the cache distribution bias).

#### 5.1.2. Learning the Scalars and Optimizing the Selection Model

**State Space.** We briefly describe the composition of the state space in our model for clarity and provide the rationale for the low dimensionality of the design.

DRL-based solutions for adaptive data caching are designed based on prior knowledge which is non-existent with ACOCA [15] and nondynamic (i.e., content library). For instance, authors define state spaces as a long vector of cache state (binary matrix) or a matrix of values of performance matrices (e.g., relative popularity) for each content. Apart from the fact that a similar definition for ACOCA can result in an exponential growth in the state space for each novel context information inferred, large state spaces are typically inefficient to train, which, as well, is contrary to our goal of fast convergence [21]. Further, a large sparse state space with some context information being accessed only once in a long time or never, redundantly occupying memory and processing is unsuitable for a CMP designed for cost efficiency and light weightiness [21].

The $s_{t}$ is the state space for a given decision epoch *t*, where $s_{t}$ is defined to provide a snapshot of the system state using the (a) total size of cached context, (b) cost of caching, (c) earnings from responding to context queries, (d) penalties incurred, (e) retrieval cost, (f) processing cost, (g) probability of delay, (h) hit rate, (i) average cache lifetime, and (j) average delay time.

**Action Space.** Action spaces in the previous literature are defined either as (a) a binary vector where indices represent the content and the binary value represents the action to cache or not cache or (b) a vector with the size of the number of slots in the cache, the values of which represent the index or identifier of a content to cache. Given we opted to generalize the state space by system parameters, the action space given by $a_{t}=\varphi$ + $\left\{\theta \right\}$ sets the parameters for the hyperplane that maximizes the separation between cost and performance efficient context to cache and not cache. Therefore, $a_{t}$ is a fixed-sized vector.

**Reward function.** The reward used in this process is the gain acquired from responding to context queries per second in the last decision window for the set φ + θ as indicated in (24) and (25). We expect the weights to converge to a value while θ does so also, resulting in a uniform distribution of *conf* values and rewards to converge to a maximum value. In (24), there are two parameters, Earning and $Pen_{ret}$, referring to monetary income earned by the CMP. Earning is the direct income for responding to context queries in adherence to all quality parameters set in the SLA. $Pen_{ret}$ is the consequential income made by the CMP as a result of nonquality adhering CPs, such as when CPs exceed the $RetL_{max}$ in SLA. The $Pen_{ret}$ from all CPs (c∈C) is given in (26), where Pen% is the percentage from the cost per each retrieval. The other parameters are costs incurred when responding to a context-query-processing cost, context-storage cost, context-cache cost, context-retrieval cost, and penalty costs. Equation (24) is an extension of *Gain* in [11].

(24) $$Gain=Earnings+Pen_{ret}-Cost_{process}-Cost_{penalty}-Cost_{storage}-\;Cost_{cache}-Cost_{retrieval}$$

(25) $$Reward=\bar{Gain}=\left(Gain-Pen_{ret}\right)/Throughput$$

(26) $$Pen_{ret}=\sum_{c=1}^{C}\left\{\left(E\left[RetL_{c}\right]-RetL_{max,c}\right)\times Cost_{ret,c}\times Pen{\%}_{c}\times E\left[Retrievals_{c}\right]\right\}$$

(27) $$Pen{\%}_{c}=\left\{\begin{array}{c} 0;\;when\;E\left[RetL_{c}\right]\leq \;RetL_{max,c}\;\\ as\;defined\;in\;the\;SLA;otherwise \end{array}\right.$$

Note that the $Pen_{ret}$ is earned for each time unit (i.e., each second in our case) that is elapsed beyond $RetL_{max}$. Therefore, CPs that are consistently unable to meet the quality parameters can be a significant form of income for the CMP which is, however, not desirable from the perspective of QoS. As a result, we consider $Pen_{ret}$ as additional earnings to the CMP and evict it from being used in any decision criteria or models in our work as shown in (26). We intend to avoid the model being biased towards retrieving from CPs that are significantly underperforming but yield a higher penalty to the CMP while the QoS of the CMP suffer.

It is important to note that the $\bar{Gain}$ in a window is affected by the evictions as well, in addition to caching because context information that has been evicted to have been refreshed proactively saves retrieval costs. Evictions are uniformly distributed against time and costs saved from evictions are offset by cached context information. Therefore, the impact of evictions on the $\bar{Gain}$ can be ignored.

**Figure 15:**
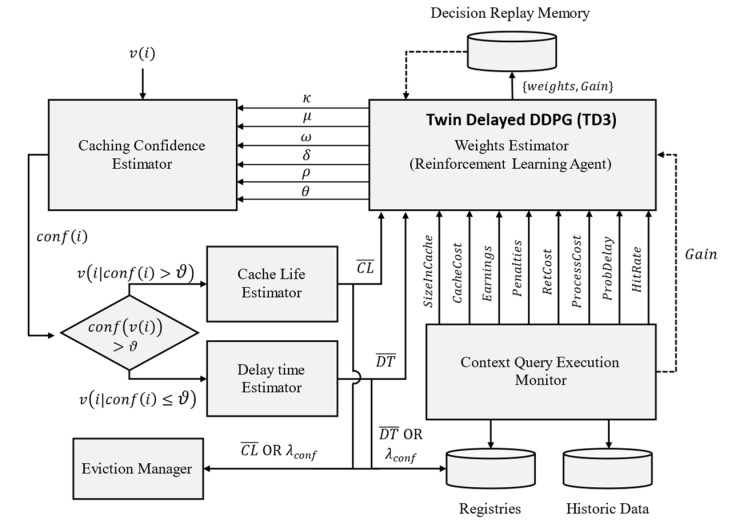
Process of solving the φ + θ set problem.

**RL-based model.** Figure 15 illustrates the process of solving this problem using a reinforcement-learning approach. RL is a suitable technique to implement in ACOCA because of three primary reasons. First, the unavailability of prior knowledge (e.g., about context and the metadata about the context-query loads) to train a model which the RL methods are capable of handling. Second, RL methods are based on continuous learning, which is suitable for adapting to subtle and/or vigorously changing environments, such as in our examples. Finally, we indicated earlier that our problem involves a multitude of parameters, either internally computed or externally provisioned. Manually designing to handle this dimensionality is extremely tedious. The relevancy of each parameter for the caching decision can vary depending on the context information being requested and/or the nature of the context-query load [15]. Deep-RL techniques provide a robust solution to handle this problem using neural networks (NNs). NNs utilize weights, biases, and activation functions to reduce the dimensionality problem internally in an adaptive manner.

We adopted the twin delayed DDPG (TD3) architecture for this purpose based on a critical evaluation of suitable adaptive reinforcement learning strategies in the literature [29,46]. Policy gradient methods, e.g., TD3 and deep deterministic policy gradient (DDPG), were attractive techniques because of the ability to parameterize the solution. Among them, we selected TD3 for our solution because the DDPG method was unstable for a significant period of time due to the variable nature of context information and the context-query load. As a result, DDPG was slower in the learning process than expected. We also tested the soft actor-critic (SAC) method as in [47] which theoretically overcomes this issue by adding an entropy value to the objective function, so that the policy does not overfit to a specific very short-term observation. In this manner, SAC is a better explorative for learning a selective context-caching agent. SAC was still underperforming in achieving short-term cost efficiency, as seen by the statistical agent (*StatAgn*), in our previous work [21] while being relatively unstable with low amounts of data and long convergence time. TD3 was viewed as a suitable alternative in this light as it learns both the Q-function and the policy concurrently, allowing better convergence. We proved this theory by executing a set of context queries under both implementations (i.e., SAC and TD3) in which TD3 quickly converged to the optimum policy. Our appendix [45] shows these comparison results for interested readers.

The context-query execution monitor (CQEM) in Figure 15 is a completely detached, independent component that continuously calculates the $\bar{Gain}$ for the weights estimator in response to the actions taken by it. $\bar{Gain}$ is affected by the caching decisions made by the caching confidence estimator based on the weights. Therefore, the cost and performance efficiencies of a CMP that is measured by the CQEM are a direct result of the φ + θ set.

The TD3 agent receives the new state $s^{\prime }$ along with the reward r=Reward calculated using (25) in the CQEM for the state, action pair $\left\{s,a\right\}$. In the TD3 architecture, there is an actor, a target actor, and two critic and target critic networks each. Using the sampled $\left\{s,a,s^{\prime },r\right\}$ set, we first derive the target action set $a_{tar}$ using the target actor network for $s^{\prime }$ and calculate the Q-values for the ($s^{\prime },\;a_{tar}$) pairs using both the target critic networks indicated by $\hat{Q}$. The following equation provides the formula for estimating the Q-value for the current policy π. The minimum of the two $\hat{Q}$ are referred to as the critical Q-value or $Q^{*}=\min\left({\hat{Q}}_{1},{\hat{Q}}_{2}\right)$ to avoid overestimating the Q-value.

(28) $$Q^{\pi }\left(s,a\right)=\sum_{s^{\prime }}p\left(s^{\prime }|s,a\right)\left[r+\gamma \sum_{s^{\prime }}(\pi (a^{\prime }|s^{\prime })\times Q^{\pi }\left(s^{\prime },a^{\prime }\right))\right]$$

Similarly, both of the critic networks are used to estimate the *Q*-values for (s,a) pairs, and then the target *Q-*value is estimated as follows:

(29) $$Q^{\pi }{}_{tar}=r+\gamma Q^{*}$$

We use the mean squared error (MSE) to calculate the loss of the critic networks against the target network and the Adam optimizer to update the network parameters. Q-values estimated from the updated critic network are used to calculate the actor loss, which is then used by the optimizer of the actor network to learn its weights. Finally, the network parameters of the target actor and the two target critic networks are updated using a soft-update process where the soft-update factor τ is configured. For instance, consider that $w_{tar}$ is a network parameter of a target-critic network and w is the same parameter of the respective critic network; then the updated parameter $w_{tar}^{\ldots }$ of the target network is:

(30) $$w_{tar}^{\prime }=\left(w_{tar}\times \tau \right)+\left(w\times \left(1-\tau \right)\right)$$

In the above process, {$s^{\prime },r$ } in the $\left\{s,a,s^{\prime },r\right\}$ tuple is appended to $\left\{s,a\right\}$ one learning epoch after adding the former. There are two reasons for this design choice: (a) the action set is applicable only during the following epoch where the weights are used to make the adaptive context-caching decisions; hence, $s^{\prime }$ and r are functions of s and a, and (b) to derive the actual reward from taking the action set rather than estimating the reward as done in the previous literature. Given the actor and critic networks learn using mini batches of the latest decision history from the decision replay memory, the delayed completion is ignorable for a continuously converging system.

In order to avoid the TD3 agent converging deterministically, we introduced an adaptive noise to the actions using a random normal distribution where μ=0 and $\sigma^{2}$ is variable. When ΔReward→0 in a learning epoch, $\sigma^{2}=\sigma^{2}-\Upsilon$ bound to a minimum of zero or else $\sigma^{2}=\sigma^{2}+\Upsilon$ bound to a maximum of 0.5. To further ensure the model does not converge prematurely to a deterministic state and overcome the cold-start problem to a certain extent, we introduced a warmup period $T_{warmup}$ during which time the model is fully explorative.

It is noteworthy to mention the rationale to feature engineering and define a small scale-state and action space. In Section 1 and Section 3, we argued about exploding state and action spaces to contribute to the exploding cost of the adaptation problem. Therefore, the limited state space using limited dimensions mitigates this problem, among others described later in the paper.

### 5.2. Access Trend

The context-query execution monitor, referred to as the CQEM, observes, stores, and aggregates all the performance metrics at different abstraction levels of the CMP—the overall CMP level, component level, method level, and context level. These performance metrics are recorded as time series using TimeScale DB (https://www.timescale.com) which allows us to produce time series-based estimations. Since the access rate of a piece of context information (AR) is one of these metrics, we estimate the trend of the AR (referred to as the access trend (AT)) using time-series regression.

Considering g:t→R is the time series-based regression function learned from the observed AR for a context *i*, $E\left[AT\right]=g\left(t^{\prime }\right)$ where $t^{\prime }>t$. As in the previous work [21,38], AT is an indicator of the popularity of a context (or a class of context), which is shown to be positively correlated to the hit rate (HR) of the item if cached based on previous literature [3,4].

### 5.3. Cache Efficiency

Cache efficiency (CE) measures the ratio between cache memory-related costs when resolving a context query using redirector mode versus using cached-context information. CE provides an estimate of how expensive it is not to cache compared to retrieving from the context cache. A high value for the CE indicates the context information is less expensive to store in the context cache than to retrieve from the CP(s). This definition is provided in (31) where $Cost_{cached}$ is the cost incurred to the CMP if the item is cached, $Cost_{redir}$ is the cost incurred to the CMP if the item is not cached (i.e., using redirector mode (*redir*)). In (32), $Cost_{cached}$ is the sum of occupied cache space cost, processing cost of retrieval and looking up for a partial miss, and processing cost to look up and retrieve from the cache when hit. In (33), $Cost_{redir}$ is the sum of processing costs for retrieval and lookup resulting in a full miss.

(31) $$CE\left(i\right)=\frac{Cost_{redir}}{Cost_{cached}}$$

(32) $$Cost_{cached}=Size\times Cost_{caching}+(Cost_{process}/W)\times \left[\left(\left(OH_{partialmiss}+\;E\left[RetL\right]\right)\times E\left[MR\right]\right)+\left(OH_{hit}\times \left(1-E\left[MR\right]\right)\right)\right]$$

(33) $$Cost_{redir}=\left(Cost_{process}/W\right)\times \left(E\left[RetL\right]+OH_{fullmiss}\right)$$

(34) $$Cost_{process}={\left(No\;of\;Mn\;Instructions\right)}_{w}\times Cost\;per\;Mn\;Instructions$$

(35) $$Cost_{process}=CPU\;miliseconds\;used\;for\;cache\;operations\times \;Cost\;per\;CPU\;milisecond$$

where *Size* is the physical size of the context in bytes, $Cost_{caching}$ is the cost of caching a byte in the context-cache memory, $Cost_{process}$ is the expected total cost of processing to be incurred during the current learning window, W is the size of the window in seconds, $OH_{partialmiss}$ is the latency overhead of looking up in the hash table of the context cache memory in the event of a partial miss, $OH_{fullmiss}$ is the latency overhead of looking up in the hash table of the context-cache memory in the event of a complete miss (i.e., not cached), $OH_{hit}$ is the latency overhead of looking up and reading from the cache memory for a hit, $E\left[MR\right]$ is the expected miss rate for the item if cached (considering (41)), and $E\left[RetL\right]$ is the expected retrieval latency (estimated from time series projection) of the context information. $Cost_{process}$ in (34) and (35) are the costs incurred by the CMP to perform the cache-related operations (i.e., lookup, read, and refresh) and can be calculated either as in (34) or (35), depending on the SLA of the cloud provider (e.g., the Cost per CPU milisecond using AWS EC2 is up to AUD 0.0000024).

### 5.4. Retrieval Efficiency

The retrieval efficiency (RE) calculates the ratio between the retrieval-related costs when responding to a context query with the context information in consideration using the redirector mode ($Cost_{\left(ret|redir\right)}$) versus it being cached and refreshed ($Cost_{(ret|cached)\;}$). This is mathematically defined in (36). RE provides a relative estimate of how much the CMP would incur on retrieval compared to having the context in the cache.

(36) $$RE\left(i\right)=\frac{Cost_{\left(ret|redir\right)}}{Cost_{(ret|cached)\;}}$$

Equations (37)–(41) below indicate the derivations of the components of (36) where, $E\left[RetCost\right]$ is the expected cost of retrieving the context based on the time-series prediction, $E\left[AR\right]$ is the expected AR of the item based on time series projection, $E\left[retL\left(i\right)\right]$ is the expected retrieval latency of the item based on time series projection, $E\left[PenaltyCost\right]$ is the expected cost of penalty per delayed context-query response based on the time-series projection and probability distribution of consumer SLAs applied on the context, $E\left[RT_{max}\right]$ is the expected maximum-accepted response latency for the context consumers based on the time-series projection and probability distribution of consumer SLAs applied on the context queries that access the item, $E\left[f_{thr}\right]$ is the expected minimum freshness for the set of context information (set by the context consumers) that is estimated based on the time-series projection and probability distribution of consumer SLAs applied on the context queries that access the item, $P\left(Delay|i\right)$ is the probability of delay, the chance of responding to a context query that accesses the context information resulting in responding to the query later than the $E\left[RT_{max}\right]$, *SI* is the sampling interval of the context provider, *L* is the lifetime of the context, and *RR* is the refresh rate once the item is cached.

$Cost_{\left(ret|ret\right)}$ is the sum of all retrievals per context query that requested context information and any penalty costs incurred for not meeting the timeliness requirements (induced by the retrievals). $Cost_{\left(ret|cached\right)}$ is the sum of refreshing cost and any applicable penalties (depending on the probability of delay [21]).

(37) $$Cost_{\left(ret|redir\right)}=\left(E\left[RetCost\right]+E\left[PenaltyCost\right]\right)\times E\left[AR\right]$$

(38) $$E\left[PenaltyCost\right]=\left\{\begin{array}{c} E\left[Penalty\right];E\left[retL\left(i\right)\right]\geq E\left[RT_{max}\right]\\ E\left[Penalty\right]\times PD;otherwise \end{array}\right.$$

(39) $$Cost_{\left(ret|cached\right)}=\left\{\begin{array}{c} \left(E\left[RetCost\right]+E\left[PenaltyCost\right]\right)\\ \times E\left[AR\right]\times E\left[MR\right];aperodic\;sampling\\ \left(E\left[RetCost\right]+E\left[PenaltyCost\right]\right)\times RR\\ ;otherwise \end{array}\right.$$

(40) $$RR=\left\{\begin{array}{c} \frac{1}{SI+\left(\left(L-SI\right)\times \left(1-E\left[f_{thr}\right]\right)\right)};L>SI\\ \frac{1}{SI};otherwise \end{array}\right.$$

(41) $$E\left[MR\right]=\left\{\begin{array}{c} \frac{1}{\left[1+\left(E\left[AR\right]\times \left(L-E\left[retL\left(i\right)\right]\right)\times \left(1-E\left[f_{thr}\right]\right)\right)\right]}\\ ;aperiodic\;sampling\\ \frac{1}{\left[1+\left(E\left[AR\right]\times \left\{SI+\left(L-SI\right)\times \left(1-E\left[f_{thr}\right]\right)\right\}\right)\right]}\\ ;L>SI\\ \frac{1}{1+\left(E\left[AR\right]\times SI\right)};otherwise \end{array}\right.$$

The probability of delay is calculated using a z-distribution. We develop the probability distribution of retrieval latencies ($P(RetL\left(i\right)\sim N\left(\bar{RetL,}\sigma_{retL}\right)$) of a context information using the CQEM, which is then transformed to a z-distribution ($Z\sim N\left(0,1\right)$). Considering $E\left[RT_{max}\right]$ as the threshold, we can derive the probability of delay from the z-distribution considering the area under the curve.

As we have indicated in Section 5.1, we ignored $Pen_{ret}$ in $RE\left(i\right)$. It avoids the decision criteria being biased towards caching context information that could yield greater earnings from penalties but suffer in QoS when the total earnings from penalties are significantly greater than $E\left[PenaltyCost\right]$ incurred by the CMP.

A high value of RE typically indicates that the context is more efficient to retrieve from and manage in the cache memory than to retrieve from the context provider. As we showed in several examples before, certain context information may not be efficient to be cached, such as when the context has a shorter *ExpPrd* than inter-request interval.

### 5.5. Un/Reliability of Retrieval

The reliability of retrieval (*Reli*) is the probability to retrieve a context in the first attempt. Then, the unreliability of retrieval (*Unreli*) = *1–Reli*. Our algorithm allows us to reattempt a retrieval of a context either from the context provider or the derivation process (depending on the logical level of the context). *Unreli* is mathematically defined in (42). Currently, the maximum number of attempts is randomly set at twenty (but configurable) for testing. If the context cannot be derived during this number of attempts, the algorithm moves on to retrieve the context from the next-best context provider or the context derived from the retrieved data from the next-best provider (if the context is retrieved from a process).

(42) $$Unreli\left(i\right)=1-\frac{No\;of\;Successful\;AND\;Timely\;Retrievals}{Total\;No\;of\;Retrievals}$$

(43) $$\bar{RetL\left(i\right)}=\frac{\sum_{s=0}^{S}retL_{s}+\sum_{f=0}^{F}retL_{f}}{S}$$

As a result, when S→0 then $\bar{RetL\left(i\right)}\to \infty$. Considering RetLi¯1∝Relii, $Reli\left(i\right)\to 0$ ($Unreli\left(i\right)\to 1$) as well.

Reliability is implicitly captured in $E\left[retL\left(i\right)\right]$ when deriving the $RE\left(i\right)$. Since $RE\left(i\right)$ is a ratio value, the impact of *Reli*(*i*) is nullified. As a result, *Unreli* is an important parameter for the caching decision. For example, it is potent to cache an unreliable context so that the chance of exceeding the *RT_max_* is minimized as caching overcomes the large latency of unreliably retrieving (with reattempts) from the context provider or the process.

### 5.6. Expected Complexity of Context Queries That Use the Context to Respond to a Context Query

The complexity of context queries differs depending on the situations and scenarios. Using the example context queries below, we can illustrate that for the same scenario, car park recommendation, there can be context queries of different complexities. As a result, even though a context may be reused among these context queries (either of the same scenario or different), the effectiveness of retrieving from the cache can be different. For instance, the performance benefit of accessing a context from the cache rather than from the context provider positively correlates with the complexity of the query [1]. Therefore, we use the complexity of the context query that accesses the item as a parameter in our equation. By extending the Halstead’s complexity theory suitably for CDQL, the complexity of a context query is calculated as follows:

(44) $$Cmpx=\frac{N\left(\left\{Operands\right\}\right)}{2}\times \frac{N\left(Operators\right)}{N\left(Operands\right)}$$

where {*Operands*} is the set of unique operands in the context query, *Operators* is the collection of all operators used in the context query, and *Operands* is the collection of all the operands in the context query. We consider context functions in CDQL [48] as a type of operator and all contexts used in deriving the result from the context functions are to be also considered in the set of operands.

**Figure 16:**
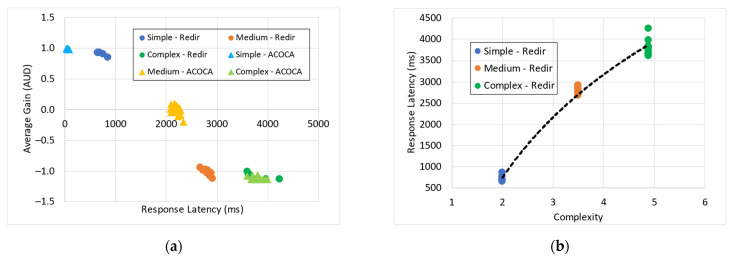
(**a**) $\bar{Gain}-RT$, and (**b**) RT-Query complexity graphs for context-query loads of different complexities.

Consider the following three context queries as examples from our motivating scenario. Table 4 summarizes the parameters of the equation. As expected, context queries are arranged in the order of complexity. We pre-emptively tested homogenous context loads of each complexity to verify our rationales. Figure 16 shows the performance of the CMP in executing those query loads in redirector mode (*Redir*), where we observed that the need for caching context increases proportionately to the complexity of the query.

prefix schema:http://schema.org pull (targetCarpark.*) define entity targetCarpark is from schema:ParkingFacility where targetCarpark.isOpen = true and targetCarpark.availableSlots > 0 and targetCarpark.price <= ${PRICE}**Query 1.** Context query of low complexity (will refer to as the “Simple” query).prefix schema:http://schema.org pull (targetCarpark.*) define
entity targetLocation is from schema:Place
where targetLocation.name=“${ADDRESS}”, entity consumerCar is from schema:Vehicle where consumerCar.vin=“${VIN}”, entity targetCarpark is from schema:ParkingFacility where

distance(targetCarpark.location, targetLocation.location) < {“value”:${DISTANCE},”unit”:”m”} and targetCarpark.isOpen = true and targetCarpark.availableSlots > 0 and targetCarpark.rating >= ${RATING} and targetCarpark.price <= ${PRICE} and targetCarpark.maxHeight > consumerCar.height**Query 2.** Context query of higher complexity to Query 1 (will refer to as the “Medium” query).prefix schema:http://schema.org pull (targetCarpark.*) define
entity targetLocation is from schema:Place where targetLocation.name=“${ADDRESS}”, entity consumerCar is from schema:Vehicle where consumerCar.vin=“${VIN}”, entity targetWeather is from schema:Thing where targetWeather.location=“Melbourne,Australia”, entity targetCarpark is from schema:ParkingFacility where
 ((distance(targetCarpark.location, targetLocation.location, “walking”) < {“value”:${DISTANCE},”unit”:”m”} and
goodForWalking(targetWeather) >= 0.6) or goodForWalking(targetWeather) >= 0.9) and targetCarpark.isOpen = true and targetCarpark.availableSlots > 0 and targetCarpark.rating >= ${RATING} and targetCarpark.price <= ${PRICE} and targetCarpark.maxHeight > consumerCar.height**Query 3.** Context query of higher complexity to Query 3 (will refer to as the “Complex” query).

Since context information is reused among context-query responses and derived higher-level contexts, it is not fair to consider the access of a context for a specific context query at the decision time. In order for the context to be cost effective, it has to be usefully accessed for responding to a number of context queries. We use the notion of context-query classes for this matter to capture generalized expected access to context concerning a range of context queries that may access them. Further details about context-query classes will be provided in the next subsection. Therefore, considering *J* as the set of context-query classes that access a context, and $P{\left(i\right)}_{j}$ is the probability of using the context in responding to the context queries belonging to this class *j*, (45) indicates the equation to derive the expected complexity of the context queries that access the context *i*, denoted as *Cmpx(i)*.

(45) $$E\left[Cmpx\left(i\right)\right]=\sum_{j=0}^{J}\left(\frac{N\left(\left\{Operands\right\}\right)}{2}\times \frac{N\left(Operators\right)}{N\left(Operands\right)}\times P{\left(i\right)}_{j}\right)$$

### 5.7. Context-Query Classes

We propose several strategies to mitigate the exploding cost of the adaptation problem in this paper. For clarity, they are as follows:

- small-scale state and action space defined using low dimensions;
- time-aware context-cache residence (i.e., estimated cache lifetime-based eviction) and latent decision-making (i.e., using the estimated delay time);
- adaptively select and switch between refreshing policies to minimize the overhead of refreshing;
- identify and aggregate performance monitoring of context to minimize the overhead of monitoring individual context.

Context-query class (CoQC) is a distinct set of semantically similar context queries. The distinction occurs as a result of the differences between the entities, conditions, context functions, attributes, etc. used to define the context queries. For example, context queries to get car-park recommendations are defined differently from context queries that subscribe to get notified of impending hazards for bikers or pedestrians. The latter also has a higher quality demand (e.g., higher *f_thr_*). Therefore, the distribution of applicable consumer SLA parameters can differ significantly. CoQC is distinctive from the clustering approach in [27] which only identifies context entities by their types (e.g., cars and car parks). The major drawback of this approach to clustering in [27] is the inability to predict the reusability of context information for previously unseen context queries, as this approach ignores the semantic similarities of using that which the lack of prior information for selective caching decision-making can be overcome.

- a discretization technique also used to reduce the computational complexity of having to monitor, maintain records, and perform calculations individually for each independent piece of context information;
- generalize the performance data over a similar set of queries so that the learned model does not overfit;
- useful in collaborative filtering to cache novel context information that are not previously observed (and profiled) when making caching decisions based on similarity.

**Figure 17:**
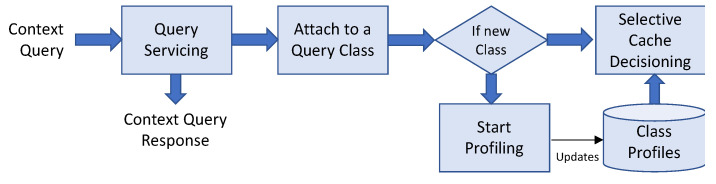
Process of recognizing context-query classes.

We opt for online clustering to identify query classes in near realtime using the context-query parse tree. Similar work with relational queries can be found in [49,50]. Each of the context-query classes is attached with expected performance values (e.g., $E\left[retL\right]$), and SLA parameters (e.g., $E\left[f_{thr}\right]$) in each learning cycle (i.e., at the end of each learning window). The process of recognizing context-query classes is indicated below in Figure 17.

**Figure 18:**
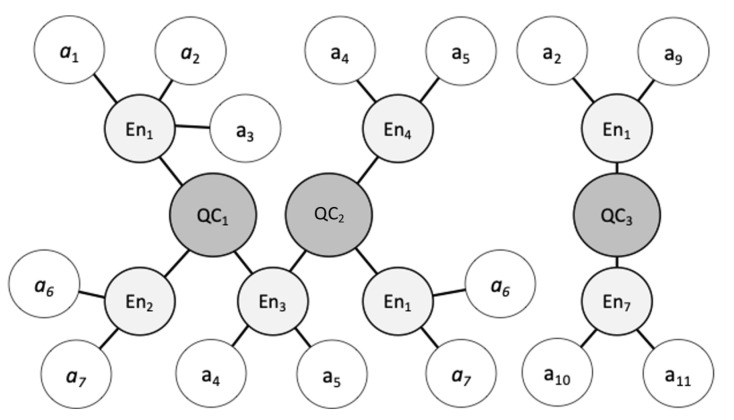
Generic example of a learnt context-query class set.

Figure 18 illustrates a generic example of three recognized query classes—QC_1_, QC_2_, and QC_3_. All edges in the graph refer to a “belongs to” relationship, e.g., a_1_ belongs to En_1_ and En_4_ belongs to QC_2_. According to (45), the expected complexity is a function considering all the relevant context-query classes that the item may be accessed from. Therefore, if *a*_1_, a context attribute derived from a certain context provider in multiple context entities (i.e., En_1_ and En_3_), is to be evaluated for caching, then we have to consider the complexity of all three query classes.

Further details about the implementation of the context-query class identification, clustering, and updates will not be discussed as it is out of the scope of this paper.

### 5.8. Estimating Cache Lifetime

Based on our previous work [11], we developed a hybrid time-aware hierarchical least frequently used eviction algorithm to manage context evictions from the cache. The estimated cache residence time also dubbed the cache lifetime (CL) in this paper, provides a means of guaranteeing a minimum CL for the context in cache memory. This policy provides several advantages, but primarily (a) maximizes the cache occupancy of limited-sized cache memory or stateful cache-memory instances, increasing the context cache utility and (b) avoids premature evictions (i.e., evicting the most lately cached items) which are otherwise characteristic of the least frequently used (LFU) policy.

It is straightforward to estimate the cost of refreshing ($Cost_{ref}$) using (47) for cached context as follows:

(46) $$Cost_{ref}=RR\times Cost_{ret}\times CL$$

(47) $$\bar{Cost_{ref}}=\frac{\sum_{i=0}^{I}RR_{i}\times Cost_{ret,i}\times CL_{i}}{I}$$

Traditional data caching (i.e., the cache-all policies), and even the context-aware caching policies in the literature, are not time-aware, resulting in CL→∞; hence, $Cost_{ref}\to \infty$. According to (39), when the retrieval inefficient context gets cached either by popularity (using the context-aware caching policies), or the traditional cache-all policies, $RR_{i}\to \infty$, and/or $Cost_{ret,i}\gg Earning$ from the context-query response in which the context is used. Assuming that all the contexts are similarly sized and therefore would result in, at most, *I* number of contexts being cached at a given limited sized cache, $\bar{Cost_{ref}}$ increases exponentially with respect to the number of retrieval-inefficient context cached.

**Figure 19:**
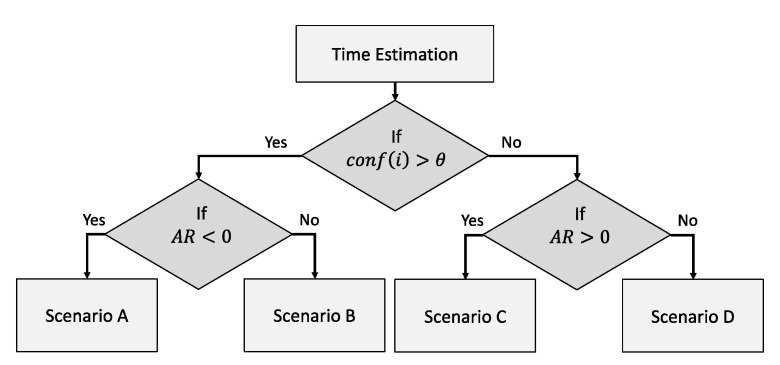
The four scenarios of CL and DT estimation.

Figure 19 indicates the scenarios in which the CL and the delay time (which will be discussed in the next subsection) will be estimated. The $trend\left(AR\right)$ is calculated using linear regression based on historical-performance data concerning the context of interest for selective context-caching selection. Accordingly, there are two scenarios: (a) scenario A, and (b) scenario B where CL is estimated.

**Figure 20:**
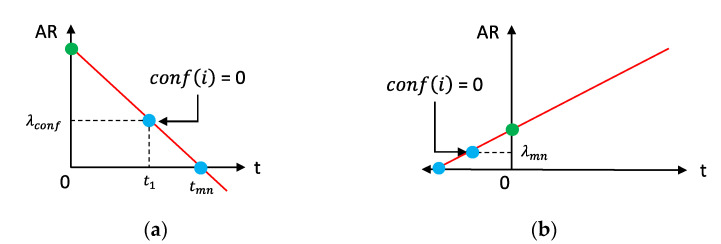
Solving the (**a**) definite CL when $trend\left(AR\right)<0$ and $conf\left(i\right)>\vartheta$ and (**b**) indefinite CL when $trend\left(AR\right)\geq 0$ and $conf\left(i\right)>\vartheta$.

In scenario A, as depicted in Figure 20 (a), $CL=\min\left(t_{1},t_{mn}\right)$. We first solve the AR when $conf\left(i\right)=0$, $\lambda_{conf}\;$as indicated below, by modifying (14) using (15)–(41) when $conf\left(i\right)=0$; the decision criteria to evict a piece of context information from the cache since it would no longer be efficient to cache. *Cmpx* of a context query and CE of context are constant irrespective of the time. Assuming ΔAT→0 for a foreseeable period of time such as in a planning period [12] and ΔUnreli→0, similarly considering the CP does not change, then the parameter that drives $conf\left(i\right)$ to zero is RE. Considering $E{\left[RE\right]}_{0}$ is the expected RE when $conf\left(i\right)=0$, $1/E\left[AR\right]=\lambda_{conf}$ can be derived as in (49) by solving (20) for $E{\left[RE\right]}_{0}$. If $\lambda_{conf}>0$, $CL=t_{1}$ is solved using the linear equation in (50) or the context information is cached indefinitely until the minimum request rate for efficiency $\lambda_{mn}=\lambda_{conf}$ is reached.


(48) $$E{\left[RE\right]}_{0}=\frac{-\left(\kappa E\left[AT\right]+\mu E\left[CE\right]+\delta E\left[Unreli\right]+\rho E\left[Cmpx\right]\right)}{\omega }$$

(49) $$\lambda_{conf}=\left\{\begin{array}{c} \frac{1}{E{\left[AR\right]}_{0}}=\frac{E{\left[RE\right]}_{0}}{RR};periodic\;\\ 0\;;\;aperiodic \end{array}\right.$$

(50) $$\lambda_{conf}=\left(trend\left(AR\right)\times t_{1}\right)+E\left[AR\right]\;t_{1}=\frac{\lambda_{conf}-E\left[AR\right]}{trend\left(AR\right)}$$

CL can be definite (i.e., as estimated above) or indefinite depending on $trend\left(AR\right)$ and $conf\left(i\right)$. Indefinite CLs refer to caching a context for an indefinite period of time until an indirect condition is satisfied that it would no longer be cost efficient to cache the item. We use the AR as the indirect condition since it is imperative that a cache item will not be cost effective to cache based on our theories discussed involving AR, lifetime, retrieval latency, and $f_{thr}$. As depicted in Figure 20b a context will be cached at least until the AR for the context reaches $\lambda_{mn}=\max\left(\lambda_{conf},0\right)$.

The probability of caching a context longer than it is cost efficient to cache ($P\left(CL_{ce}<CL_{actual}\right)$), given it is cached indefinitely, would be zero because the continuous monitoring mechanism of ACOCA evicts the context as soon as the cost-inefficient conditions are met. On the other hand, we minimized $P\left(CL_{ce}<CL_{actual}\right)$ by calculating the definite $CL=\min\left(t_{1},t_{mn}\right).\;C$ontext consumers make queries for context in relation to a situation, event, scenario, or scene, which is time progressive [51]. The popularity of a topic is typically sixty minutes [3]. We constrained the estimated $CL\in \left(0,3600\right]$ in seconds. Therefore, when CL<60 mins, then $P\left(CL_{ce}<CL_{actual}\right)\to 0$ because we assume that ACOCA does not incur additional costs of context-cache management due to overestimating CL.

### 5.9. Estimating Delay Time

The processes of adaptation for adaptive context caching are an additional overhead when compared to a nonadaptive strategy. The cost of additional processing is absorbed in the gain or loss of calculation of responding to context queries according to (24) in $Cost_{process}$. In order to minimize the cost of responding to context queries, we introduce the delay time (DT)—a wait for a context until it will be re-evaluated to be selectively cached.

DT is estimated only after a context is evaluated not to be cached in the context cache memory when $conf\left(i\right)\leq \vartheta$ under the same conditions. Our estimation of DT calculates the probable time until which the context *I* is expected to produce a $conf\left(i\right)>\vartheta$. In other words, DT is an estimation of the time at which the context information is expected to be cost and performance efficient to be cached. A similar analogy to our rationale in the traditional data-caching policies can be found with Belady’s optimal replacement algorithm [52] where the pages whose next use is expected to be furthest in the future are replaced. In comparison, our estimation technique is not only usage aware but also cost and performance-efficiency aware when deferring the caching decision.

Although the weights are updated frequently after each learning cycle which can result either in a reduction or increase in the $DT_{actual}$ in between the time DT is estimated and elapsed, the delay is still advantageous for minimizing the processing overhead involved with context selection and adaptation. For instance, if $DT_{actual}$ < DT, the $\Delta Gain=Gain_{redir}-Gain_{cache}>0$ during this time because the context can be justified to be not cached due to a lack of confidence to cache; ΔGain≤0 otherwise, but we assume $DT\to DT_{actual}$ because we estimate the DT considering the time-series predictions and linear regression for accuracy. Therefore, the monetary regret of not caching is minimized.

We, however, introduced a maximum DT ($DT_{max}$) to minimize the number of context information being evaluated for caching due to DT→0 as cost and performance efficiency expectation of cached data is subject to time. Considering [3], $DT_{max}=60min$ is proposed in this work.

**Figure 21:**
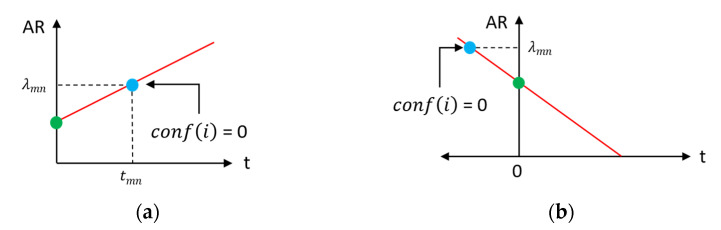
Solving the (**a**) definite DT when $trend\left(AR\right)\geq 0$ and $conf\left(i\right)\leq \vartheta$, and (**b**) indefinite DT when $trend\left(AR\right)<0$ and $conf\left(i\right)\leq \vartheta$.

According to Figure 21, DT is estimated under two circumstances: (a) scenario C, and (b) scenario D. We derive the equation given in (51) to calculate $\lambda_{conf}$ for DT, and then solve $t_{mn}$ using (49) for scenario D.

(51) $$\lambda_{conf}=\left\{\begin{array}{c} \frac{1}{E{\left[AR\right]}_{0}}=\frac{E{\left[RE\right]}_{0}}{RR};periodic\;\\ \left(\frac{Cost_{\left(ret|cached\right)}\times \omega }{E\left[MR\right]\times Cost_{\left(ret|redir\right)}\times E{\left[RE\right]}_{0}}-1\right)\times \frac{1}{ExpPrd}\;\\ ;\;aperiodic \end{array}\right.$$

### 5.10. Objective Function

In Section 1, the objectives of the ACOCA algorithm were introduced. In the following sections, we discussed and developed our theoretical models in line with our objectives. In summary, given our primary objective is to achieve a quasi-Pareto optimal state between the cost and performance efficiencies for the CMP using ACOCA where both cost and performance efficiencies are maximized, we can define the multi-objective function as follows over a time horizon *T*. CMPs are latency sensitive near realtime systems; therefore, $\bar{RT}$ is considered for minimizing. $\bar{Gain}$ represents the cost efficiency of the system which is maximized. $\bar{PD}$ is a measure of both costs and performance efficiency of the system that overall needs to be minimized. The constraints of the objectives are as follows:

- the optimization occurs continuously, subsequent to the warmup period ($T_{warmup}$);

- there should exist at least one context information accessed from the CMP.

(52) $$\underset{a}{\max}{\bar{Gain}}_{T},\;\underset{a}{\min}{\bar{RT}}_{T},\;and\underset{a}{\min}{\bar{PD}}_{T}\;subject\;to\;T>T_{warmup}\;where\;T,T_{warmup}\in R_{0}^{+}\;\exists i.AR_{i}>0$$

## 6. Evaluations

In this section, the ACOCA algorithm developed in the previous sections is evaluated against the benchmarks which will be described later. We test whether our design objectives are met and validate the theories, and rationales adopted in developing the ACOCA algorithm. We tested all the results from the benchmarks against our null hypothesis that there exists no significant statistical relationship to the results from ACOCA for a confidence level of 95% using *t*-tests. Hence, the results verified that the caching policies are independent. The errors indicated by the results conform to a 95% confidence interval.

### 6.1. Experimental Setup

**Figure 22:**
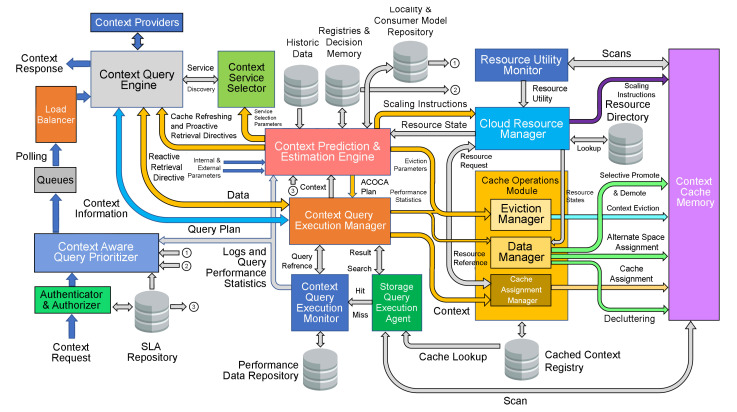
Architecture of ACOCA as integrated into a context-as-a-service platform.

ACOCA was implemented and integrated into the context-as-a-service (CoaaS) platform [7] that was implemented as a part of the bIoTope project (https://biotope-project.eu/) that was co-funded by the European Commission under the Horizon-2020 program. The implementation can be found on GitHub (https://bit.ly/reactive-acoca) Figure 22 depicts the architecture of ACOCA in CoaaS (detailed descriptions about the components and the workflow can be found in [45]). It comprises seven key components: (a) context prediction and estimation engine (CPEE), (b) context-query execution manager (CQEM), (c) storage query execution agent (SQEA), (d) query execution monitor (QEM), I cache resource manager (CreM), (f) resource utility monitor (RUM), and (g) cache operations manager (CopM). The core component of ACOCA is CPEE, which provides instructions in the form of a context cache plan [15]. The context-caching plan specifies adaptive actions such as selective context-caching instructions, cached-context-refreshing instructions, and selective-eviction instructions. The CQEM orchestrates context retrieval and writes or updates context in context storage or cache. SQEA executes context read operations in the cache for received context requests from the CQEM, and it is designed to scale horizontally using containers. The CopM is responsible for writing, updating, and eviction of context in the cache. Each cloud cache host has RUM agents installed that communicate with the CreM to scan the cache-memory instance for physical utility. The primary duty of the CreM is to perform resource adaptation for the CMP, which includes generating scaling instructions.

The reactive context-caching recommender (the subcomponent of the context prediction and estimation engine (CPREE) which performs learning for the ACOCA algorithm), is developed using Python 3.8.2. It implements the TD3 using TensorFlow 2.0 to develop artificial neural networks. The TD3 solution implements an actor network, a target-actor network, two critic networks, and two target-critic networks, each having an input layer, an output layer, and two hidden layers with 256 neurons. The Adam optimizer was used in all networks. The discount factor γ=0.9, the learning rate of the actor network α=0.001, and the critic network β=0.002. Learning occurs in mini batches of 10 recent historical decisions where the decision memory (i.e., experience replay memory) is implemented as a first-in-first-out (FIFO) storage of size 60. Soft-update factor τ=0.005 and $T_{warmup}=$ 600 s. Noisy adjustment to the actions Υ is calculated using a random normal distribution where $\sigma^{2}=0.5$.

The rest of the components were implemented using Java. MongoDB, Microsoft SQL, TimeScale, and SQLLite were used for different purposes of storage in CoaaS.

**Figure 23:**
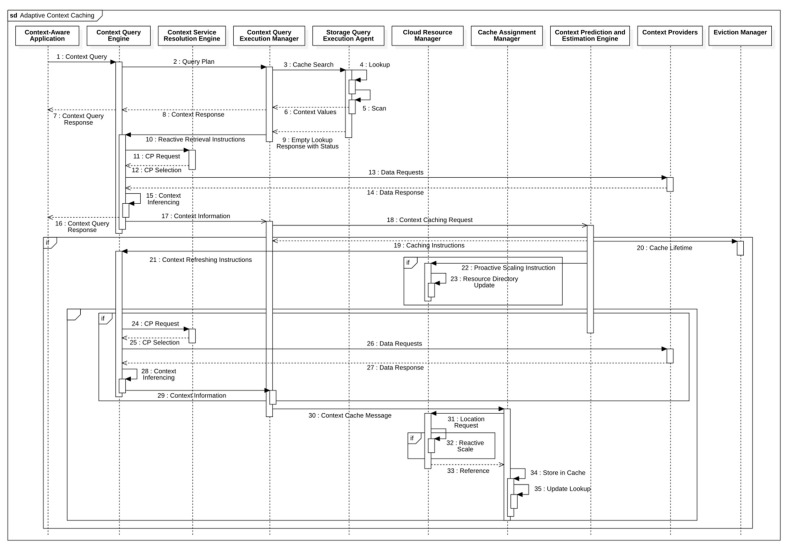
Sequence diagram on the execution sequence of responding to a context query in CoaaS using ACOCA.

Figure 23 illustrates the sequence diagram of CoaaS with ACOCA in responding to a context query. The context-query engine receives a context query which is first parsed, and then further broken down into context requests by entities. The set of context requests, referred to here as the query plan in Figure 22, is then passed to the context-query execution manager (CQEM) that coordinates the execution of each context request (CR) and, finally, aggregates their results. The CQEM directs the storage query-execution agent to perform a cache lookup. If hit, then it returns the context information for the CR. Otherwise, the CQEM invokes the context service invoker to select and retrieve data from the context providers. Depending on the nature of the context requested, the context-query engine may invoke the context resolution engine to infer context for the CR before returning the context response. If the cache miss was a partial miss, then the context derived/retrieved would be used to refresh the context in the cache via the data manager in the cache operations module. Otherwise, if the retrieval was triggered by a full miss, the CQEM directs the reactive context selection module in the context prediction and estimation engine to asynchronously evaluate the context information for caching. If the context is selected for caching, it is cached using the cache assignment manager, which also updates the hash tables.

We used Redis (https://redis.io) to implement the context cache memory because it (a) supports caching multiple different types of unstructured data and (b) provides features such as Keyspace events which are useful in ACOCA to handle definitive and indefinite cache lifetime-based eviction events. Only a single stateful instance of Redis was used during testing with a capacity of four gigabytes. In this paper, we present only the version of ACOCA which caches context entities.

The context provider (CP) simulator in [11] was extended to simulate CPs that would generate the raw context from which the context is derived for the queries. Real-world datasets were used to define the behaviour, e.g., the Melbourne weather dataset (https://www.meteoblue.com/en/weather/archive/export/, accessed on 27 February 2023) in 2021. Forty context services are simulated in our setup to access about 81,000 context providers. Each context service can be used to retrieve IoT data of a specific entity type (e.g., vehicle, car park, or location) and multiple context providers. Full specifications of the behaviours of the different context services and definitions of the parameters specified in the context provider SLA can be found in our appendix [45].

The context queries used to evaluate ACOCA were generated using the context-query generator (CQG) based on the real-world traffic conditions in Melbourne because a sizable collection of real-world context queries has yet to be collected for evaluating research in context-aware computing. Interested readers are referred to [53] for further details of the CQG. Due to the longevity of the query load that is simulated using the CQG, i.e., one week, and the inability to reproduce the same context-query load at different times of execution of our experiment, we used a random sample of 133,808 context queries from the generated context-query set (https://bit.ly/sample-context-queries, accessed on 27 February 2023) and simulated the context-query load using the Apache Jmeter. The request rate of the context queries was set to one per second, conforming to a Poisson distribution. There are ~80,900 users (i.e., commuters using the application) generating context queries in the scenario.

We proved our theories and the reactive ACOCA algorithm under two separate sections. It should be noted that the quality of service (QoS) of the CMP, which is used to measure performance efficiency is measured against the service level agreements (SLA). First, we produce and discuss the results from a scenario where the context queries originate from many users utilizing the same context-aware parking-assistance application. Only one SLA applies for all the context queries (let us refer to it as 1-SLA) as a result. It should be noted that the application user is the secondary user of a CMP, whereas the developers of these applications are primary. As a result, the quality parameters such as those defined in the SLA (considering a context-aware application as a context consumer) are predefined by the developers, coherently with the context queries they define for the application. Let the parameters of the SLA be the following:

- RT_max_ = 2 s;
- Price_res_ = AUD 1.0/timely response;
- Pen_delay_= AUD 0.5/delayed response;
- *f*_thr_ = 0.7.

We selected to set $f_{thr}=0.7$ based on the concept of feasible SLAs from [11].

Secondly, we consider a scenario where multiple context-aware applications are used to generate the context queries. Let us refer to this scenario as n-SLA. The request rate (λ) of context queries conforms to a Poisson distribution but varies over time (as in the example provided in [53]). Complexities of the context queries [45] are normally distributed where μ=2.925 and $\sigma^{2}=0.5407$. We simulate twenty context-aware applications, each having different QoC, and QoS expectations specified as feasible SLAs [11]. These SLAs were generated randomly with the feasible SLA defined in 1-SLA used as the mean. Therefore, all parameter values across all SLAs are normally distributed. Summary of the price per timely context response $Price_{res}$, freshness threshold $f_{thr}$ [11], maximum expected response latency for a context query $RT_{max}$, and penalty per context response exceeding the $RT_{max}$ ($Cost_{pen}$) of the SLAs are provided in Table 5. Each context consumer may make none or many context queries in an hour.

### 6.2. Benchmarks

The reactive ACOCA algorithm is benchmarked against the

- traditional data caching policy;
- a context-aware (popularity of context information) caching policy.

By traditional data-caching policy, we refer to the cache-all policy which is based on the write-once-read-many concept. The authors opt to least recently used (LRU) for eviction in the literature using traditional data caching which is considered as a benchmark. We hypothesize that traditional data caching is cost inefficient for caching transient context caching as it would levy a significant context-refreshing cost during the indefinite cache-residence time. For further investigation, we assigned each of our refreshing policies, reactive and proactive with shifts separately for traditionally cached context. Then, the cached context was assigned a refreshing policy resolved using our refreshing-policy selection algorithm for comparison. We refer to the aforementioned as cache all reactive, cache all proactive, and cache all resolved, respectively.

As we indicated in Section 3, context-aware caching in the literature has primarily dealt with maximizing the HR of a system [3,4,20]. Khargaria et al. [27] also implemented a context-caching algorithm based on the popularity of context entities. Therefore, all authors attempted to cache the most frequently accessed data in the cache. Caching frequently accessed context could theoretically maximize the HR, especially if the lifetime of the context is long (e.g., one of the disadvantages of [3] is caching transient data with long lifetimes despite a low rate of access, yet resulting in higher HR for the cached data). Using caching efficiency $CE\left(i\right)\;$and retrieval efficiency $RE\left(i\right)$ in Section 5 however, we argued that context with certain features, e.g. context with ephemeral lifetime and/or expiry periods, can result in cost and performance inefficiencies if cached. We highlighted in our objectives that HR is less significant compared to PD and Gain. Accordingly, not all popular contexts may be efficient to cache (we later coin the term “selective efficiency” based on this concept) which we intended to benchmark for ACOCA.

Interested readers are referred to our appendix [45] for the details of the context-aware context-selection agent developed based on previous literature. Due to the objective of maximizing HR using the popularity of context, we will refer to this agent as the popularity-based agent as well, interchangeably.

### 6.3. Results and Discussion

In this subsection, we present the observed results from the experiment and discuss how it proves the theories and policies defined and developed in Section 4 and Section 5. First, the 1-SLA scenario is discussed and then the n-SLA scenario will be discussed later.

For consistency, we use dark blue to represent the redirector mode. Orange, light blue, and pink are used to represent the traditional data-caching policies (i.e., cache all) where the refreshing policies were set to reactive, proactive, and resolved based on our algorithm, respectively. Green is used to represent context-aware caching that is based on the popularity of context information. Finally, yellow is used to represent ACOCA.

#### 6.3.1. Case with Single IoT Application (1-SLA)

We first tested our ACOCA mechanism for the 1-SLA scenario. Based on the objective function, we will present the results for improvements in QoS, cost efficiency, and overall outcomes in order.

##### Testing the Improvement in Quality of Service

**Figure 24:**
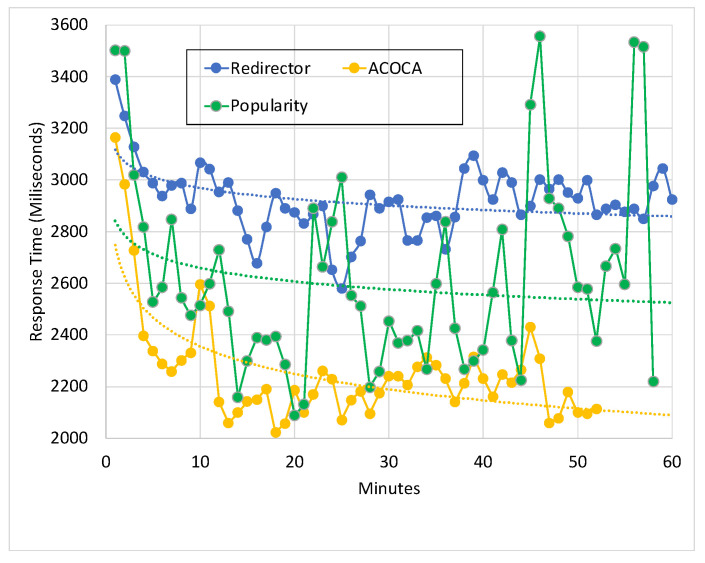
Variation of $\bar{RT}$ during the testing period.

First, we evaluated the QoS achieved by introducing ACOCA to the CMP. Figure 24 illustrates the progression of the $\bar{RT}$ during the testing period.

**Figure 25:**
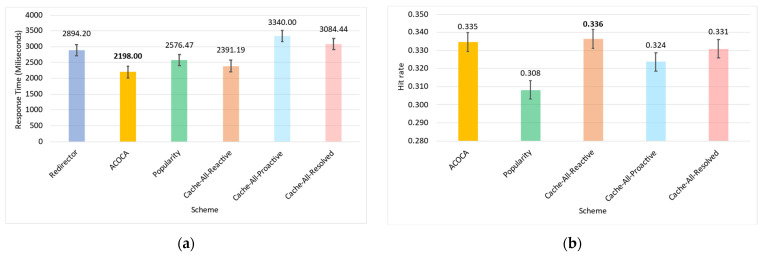
(**a**) Average throughput of the caching policies and (**b**) Overall $\bar{\mathrm{PD}}\;$ of the caching policies.

Figure 25 compares the average response times ($\bar{RT}$) and probability of delay ($\bar{PD}$). The improvements in $\bar{RT}$ were 34.2%, 14.7%, and 24%, respectively, against traditional data caching, context-aware caching, and the redirector mode. The result proves our rationale for why the traditional cache-all policies are not fully applicable when caching transient context. We find the significant process-scheduling overhead as the reason for this observation, indicated by the increase in processing costs (which we will show in the next sub-section) as the number of context entities to automatically refresh grows.

ACOCA is more performance-efficient than the context-aware caching policy because frequently accessed popular context can be cache inefficient (referring to the caching efficiency in Section 5) resulting in a lower HR than ACOCA. As we show in Figure 25 (b), the context-aware caching policy recorded the least $\bar{HR}\;$although being designed to maximize the HR, while ACOCA records an equally highest $\bar{HR}\;$among the benchmarks of 0.335±0.008. Considering that $Cost_{redir}$ remained relatively constant during testing, given that MR→1 for a number of frequently accessed contexts, and $OH_{partialmiss},OH_{hit}\ll RetL$ in (26), then the $Cost_{cached}$ increases drastically so that $CE\left(i\right)<1\;$or $CE\left(i\right)\to 0$ when Size→0 or $Cost_{caching}\to 0$, i.e., in our experiment where the cost of caching a gigabyte was costing only AUD 0.30.

Figure 25b highlights the low significance of HR in maximizing the cost and performance efficiencies of a CMP. The following are the reasons for this observation [38]:

- ephemeral lifetimes of context information;
- network latency age of context problem;
- the difference in the physical and logical lifetime of context information causes an asynchrony in context refreshing;
- unreliability of context providers.

**Figure 26:**
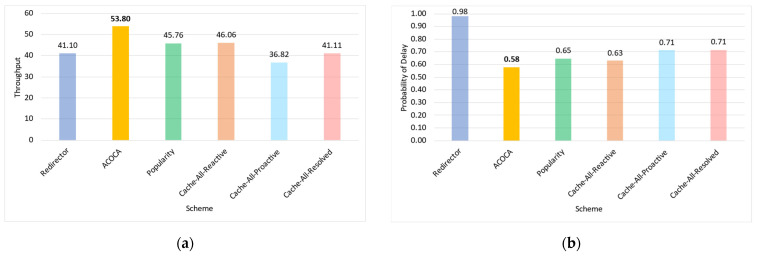
Overall (**a**) throughput of the caching policies and (**b**) $\bar{PD}$ of the caching policies.

Despite the insignificant difference in HR compared to the cache-all-reactive benchmark, the CMP recorded a higher context-query response throughput of 53.8±2.62 per minute under ACOCA (Figure 26a). ACOCA performed more retrieval operations compared to other caching policies (as we will show in the next sub-section) which is counter-intuitive given the observed HR. However, the reason for the increase in the throughput could, however, be found in (29)–(34) based on $RE\left(i\right)$, as the CMP suffers less performance degradation from slow retrievals when the context retrieval is cheap, fast, and reliable ($Reli\left(i\right)\to 1$). Note that the ACOCA recorded the lowest probability of delay (PD) 0.58±0.06 as well, which is 10.8% less than the benchmarked selective-agent context-aware caching (Figure 26b). Based on these facts, we can conclude that ACOCA is more “selective efficient” and able to cache context that is most probable to contribute positively to cost and performance efficiency. ACOCA has, therefore, learned to use context cache as a functionality to minimize PD.

**Figure 27:**
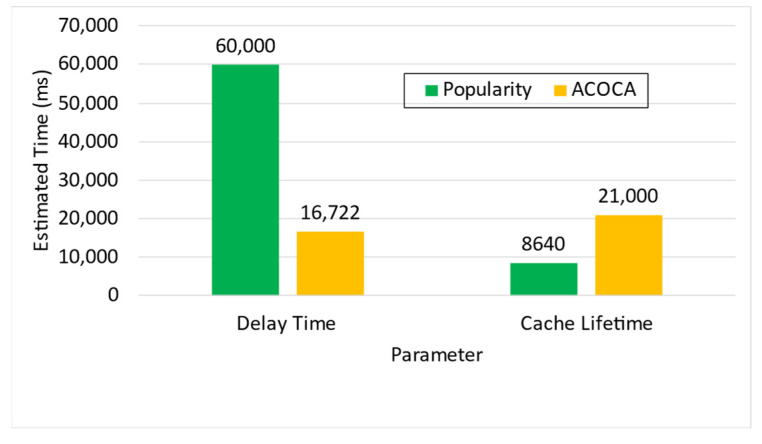
Comparison of estimated cache lifetime and delay time between ACOCA and context-aware policy.

The estimated CL and DT between ACOCA and the context-aware policy are compared in Figure 27.

ACOCA performed better in cache retention given the longer cache lifetime of context, also providing reasons for the better HR and verifying our reasoning as to why popular context may not always be the most cache and retrieval efficient. Considering the (32)–(37), estimated cache lifetime using (44)–(46) CL maximizes either when (a) $trend\left(AR\right)$ minimizes or (b) $\lambda_{conf}$ is minimized or $\lambda_{conf}\to 0$ as a result of $nonRetEff\propto RE\left(i\right)$ maximizing. Therefore, it is a clear indication that ACOCA is more selection efficient compared to context-aware data-caching techniques attempting to select a context for caching.

**Figure 28:**
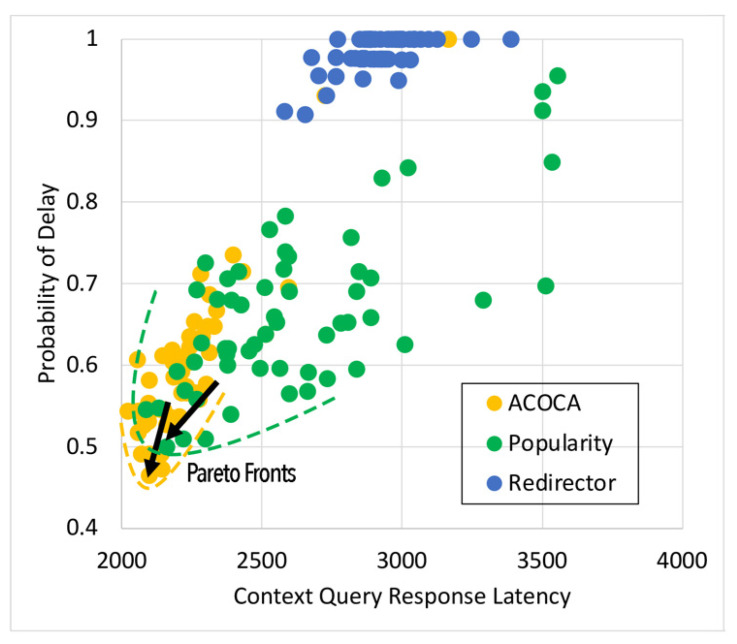
Relationship between $\bar{RT}-PD$.

Penalties due to non-performance conformant context-query responses are a critical cost incurred by a CMP, e.g. responses made later than the maximum tolerated RT for context consumers ($RT_{max}$). Therefore, the RT-PD graph provides a graphical overview of the balance of an algorithm between the cost and performance efficiencies (Figure 28).

**Figure 29:**
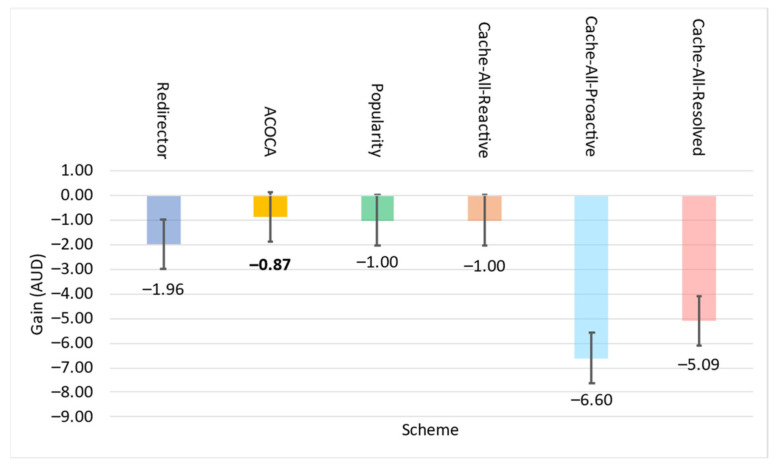
Overall $\bar{Gain}$ using the caching policies.

There are three main features in this observation: (a) the centroid of ACOCA is (2255.79, 0.61) whereas (2621.76, 0.67) for the context-aware algorithm, which indicates more cost efficiency for ACOCA, (b) ACOCA is densely clustered, i.e., the average distance from the centroid $D_{ACOCA}=134.04$, whereas the context-aware algorithm is sparse, i.e., $D_{pop}=286.10$, and (c) the redirector is densely clustered, i.e., $D_{red}=94.23$ but significantly outlying from the cached approaches. Clustered results refer to consistent performance. Since the redirector mode shows densely clustered results, we can assume the context retrieval and inferencing operations are fairly consistent as a processing overhead (POH). Therefore, the sparsity of the context-aware approach can be explained using the retrieval and caching efficiencies explained earlier. Further evidence of selection efficiency in ACOCA is self-explanatory, accordingly in Figure 29 as well. Note that the higher gain-per-context query is despite the marginally higher retrieval cost of context compared to the context-aware caching policy.

##### Testing the Improvement in Cost Efficiency

Second, overall cost efficiency was tested. ACOCA is the most cost efficient compared to our benchmarks as shown in Figure 29.

**Figure 30:**
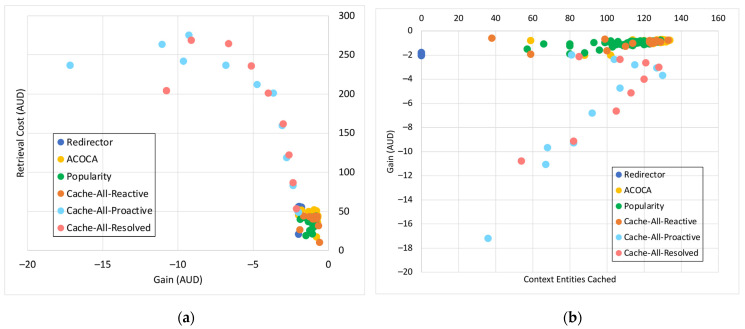
Relationship between (**a**) $\bar{Gain}-\bar{Cost_{ret}}$ and (**b**) $\bar{\mathrm{Gain}}-$Number of context entities cached.

Figure 29 and Figure 30 justify our rationale for considering lifecycle costs for adaptive context caching. The traditional caching policies have been extremely cost inefficient and we will ignore them from further discussion as an outlier to the rest of the benchmarks. We theoretically discussed this exponential cost inefficiency against selectively caching retrieval-efficient context caching in Section 5.8. For instance, cache-all-proactive and cache-all-resolved make a significantly higher number of context retrievals which is the most significant overhead for cost efficiency when context refreshing. Yet, they still performed worse among benchmarks in all the quality of service (i.e., performance efficiency) metrics.

**Figure 31:**
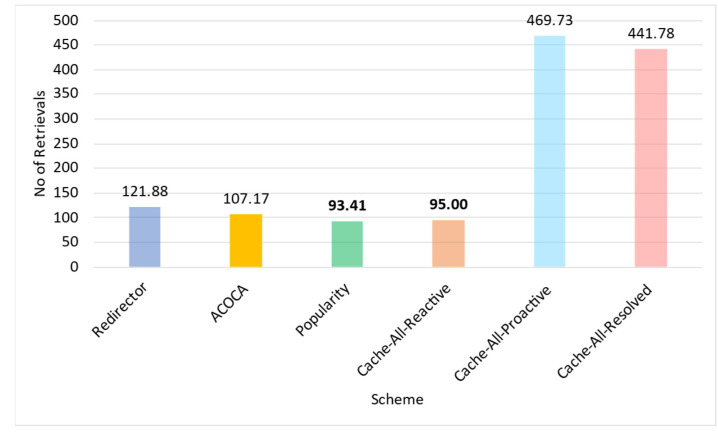
Average number of retrievals performed per window by the caching policies.

**Figure 32:**
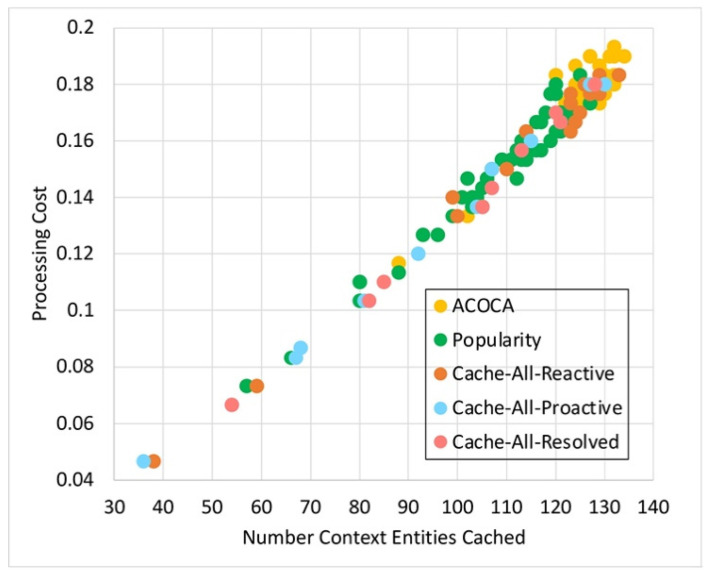
Relationship between Processing Cost–Number of context entities cached.

Figure 30, Figure 31 and Figure 32 further prove that the main cause of cost inefficiency for traditional caching policies used for transient context is context refreshing. Figure 31 and Figure 32 indicate that the CMP incurs more cost of retrieval per cached context entity than any other policy. Selecting the most efficient to cache in ACOCA using the learned $conf\left(i\right)$ increased the selection efficiency as we indicated, which resulted in a significantly smaller number of context retrievals having taken into consideration the retrieval efficiency compared to the nonselective, traditional cache-all policies.

Comparing the three cache-all traditional data-caching policies, it also is evident that not all context information can be costly, and performance efficiently refreshed using a single policy, justifying (a) resolving the most efficient refreshing policy prior to caching and (b) refreshing policy switching. In our observation, cache-all proactive results in a loss of AUD 6.60 per context query, whereas cache-all-reactive results in only a loss of AUD 1.00—at most an 86.8% cost inefficiency compared to ACOCA. We argued in Section 4 using (3)–(13), that context based on several features can be inefficient to cache and proactively refreshed despite recent work generalizing proactive refreshing to be cost and performance efficient when handling transient context [11,12]. Overall, however, given the cache-all-resolved policy is also 82.9% cost inefficient compared to ACOCA; we can conclude that the efficiencies gained in ACOCA are a combination of context selection, refreshing policy selection, and adaptive policy switching.

Given the extreme cost of context-cache management (e.g., such as the cache memory, refreshing costs, context-class clustering, and continuous monitoring of context) using the traditional cache-all policies, it is imperative that estimating and setting an expected cache life or conditions to evict using $\lambda_{conf}$ are significantly advantageous to achieve cost and performance efficiency. We theoretically developed a hypothesis for this observation in Section 5.8 and 5.9 using the probability of overestimating the cache lifetime (CL) and the processing overhead of definitely and indefinitely cached context information. In fact, Figure 30 and Figure 31 prove this hypothesis and the “exploding cost of adaptive context management” as the unmanaged benchmarks have recorded significant losses resulting from costs to the CMP compared to managed approaches such as ACOCA.

##### Testing the OVERHEAD of ACOCA to the CMP

**Figure 33:**
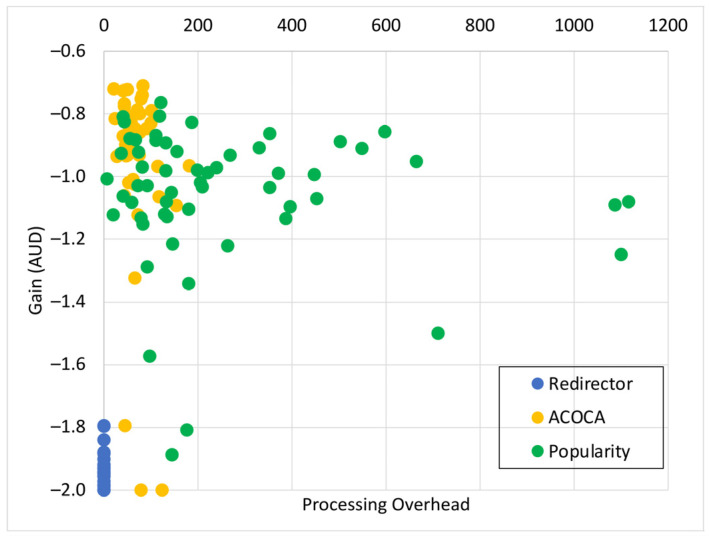
Relationship between $\bar{Gain}-POH$.

Third, we evaluated the utility of introducing the additional complexity of adaptation to the CMP. Figure 33 illustrates the relationship of the processing overhead against $\bar{Gain}$. It is clear that the adaptation results in additional processing overheads compared to the redirector mode. However, the redirector mode would incur AUD 13.84 for each second of processing, whereas ACOCA incurred only AUD 0.02 per second. Comparatively, the context-aware policy incurred only AUD 0.01 since the computational process is less complex than ACOCA, which involved a higher number of parameters and calculations. There is a 99.8% significant advantage in gain from responding to context queries for the CMP using ACOCA despite this additional processing.

We made conscious decisions in our design such as introducing definite and indefinite delay times (DT) to minimize the processing overhead involved with adaptation (as explained in Section 1, Section 2, Section 3 and Section 4). We identify this design choice as one of the contributing factors to the significant processing cost advantage. It is difficult however to benchmark this result against previous work since authors have not elaborated on the cost of adaptation in the literature. Given the typical computational expense involved with complex artificial neural networks (as our TD3 implementation), we can conclude ACOCA is computationally efficient as well.

##### Overall Outcomes for the 1-SLA Scenario

Finally, Table 6 summarizes the performance data for each of the benchmarks. CA refers to cache all in the table.

**Figure 34:**
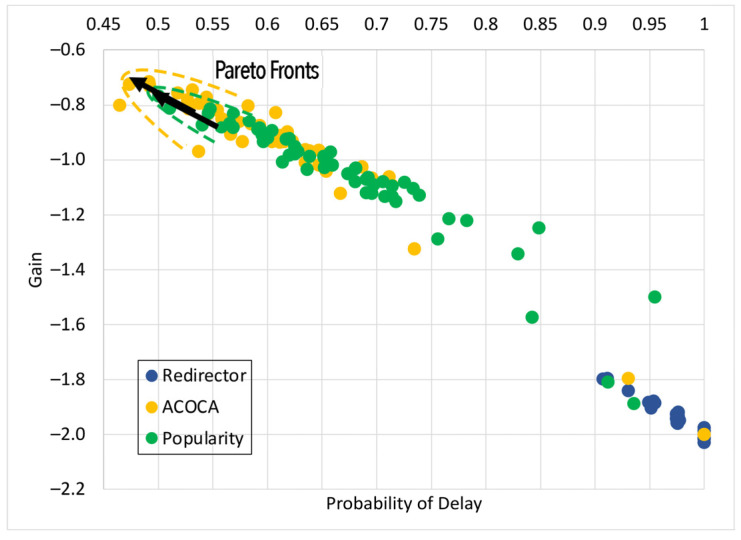
Relationship between $\bar{Gain}-PD$.

We considered three objectives for designing ACOCA in Section 1. First, the cost efficiency objective is measured using the $\bar{Gain}$ and is minimized using ACOCA among the benchmarks. Second, the objective of performance efficiency is measured using both the $\bar{RT}$ is minimized as well. The ability to respond to time-critical context queries is measured using the $\bar{PD}$ and is also minimized. Finally, we stated in Section 1 that we solved a multi-objective problem in ACOCA that should result in a Pareto optimal state. Considering that $\bar{Gain}$, $\bar{RT}$, and $\bar{PD}$ are the primary indicators of cost and performance efficiency, we can argue that ACOCA is in a Pareto optimal state that is better than the benchmarks using Figure 28 and Figure 34. We indicate this in the figures above where results could be seen closer to the (0,0) than the benchmarks, indicating the better cost and performance efficiency of ACOCA.

#### 6.3.2. Case with Multiple IoT Applications (n-SLA)

We tested our algorithm for the n-SLA scenario similarly to the previous subsection. Our algorithm against the benchmarks showed features consistent with the 1-SLA scenario and in some cases, ACOCA was significantly advantageous.

Table 7 summarizes the performance data for each benchmark. CA refers to cache-all in the table.

Accordingly, ACOCA is 67% more cost efficient compared to caching context information using context-aware (i.e., popularity-based) data-caching techniques. It is also 85.1%, and up to 95%, more cost efficient compared to the redirector mode and traditional cache-all policies, respectively.

Similar to the 1-SLA scenario, ACOCA showed the least $\bar{RT}$ and $\bar{PD}$. The CMP integrated with the traditional context-aware data-caching technique closely follows this result. The reasons could be found in the $\bar{HR}$. ACOCA showed the highest $\bar{HR}$ of 0.5316±0.00, while the traditional context-aware data caching achieved 0.5001±0.00. While the superior $\bar{HR}$ of ACOCA denote the better selection efficiency of our algorithm over the traditional context-aware policy optimized for HR (also denoted by the relative cost efficiency), the CMP was able to retrieve context information faster from the cache memory for a cache hit during our tests with traditional context-aware caching, i.e., 0.1639±0.00 ms versus 0.3586±0.01 ms. This is a result of the structural complexity of context information in the cache when selected with ACOCA.

**Figure 35:**
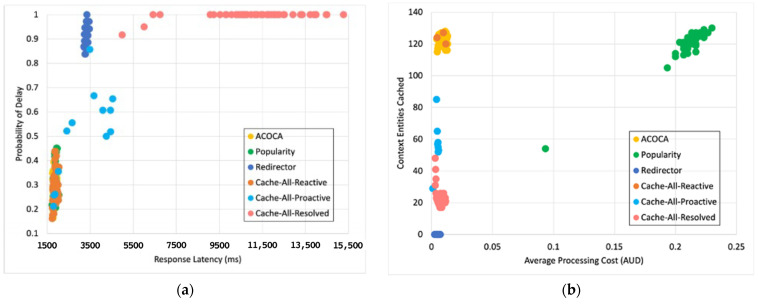
Relationship between (**a**) RT-PD and (**b**) Processing Cost–Number of context entities cached.

Figure 35a illustrates a diagram that plots the $\bar{PD}$ against $\bar{RT}$. Compared to Figure 28 above for 1-SLA, the dispersion between the clusters of each benchmark is statistically more significant. Similar is the case in Figure 35b which illustrates the relationship between the processing cost and the number of context entities cached during a window.

Figure 35b depicts two important features of the ACOCA algorithm compared to the benchmarks. First, ACOCA incurred 95.69% less processing cost compared to the CMP integrated with context-aware caching despite caching a similar number of context entities. This result experimentally proves our theory on the cache efficiency (CE), that not all frequently accessed pieces of context information are cost efficient to cache due to factors such as the holdup costs. For instance, the processing cost of a context entity using ACOCA is only AUD 0.000074. The additional computational cost introduced by ACOCA is ignorable and barely differentiable against context-oblivious caching policies such as the cache-all policies. Secondly, all cache-all policies have resulted in caching a lesser number of context entities compared to ACOCA. The context-cache memory used in our experiments is limited in size. Cache-all policies have succumbed to cache competition, which the ACOCA has alleviated by cost, size, and performance-efficiency-aware selection. Hence, ACOCA is significantly more cache-memory efficient compared to any of the benchmarks.

**Figure 36:**
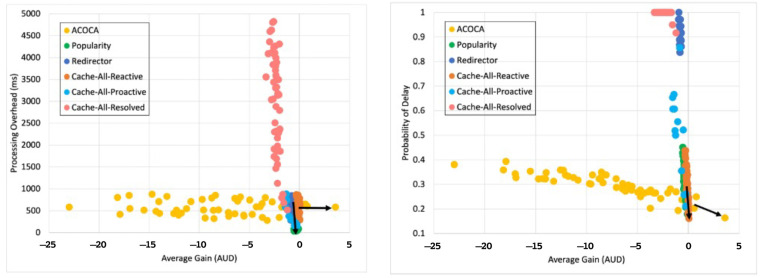
Relationship between (**a**) $\bar{Gain}-POH$ and (**b**) $\bar{Gain}-PD$.

Finally, in Figure 36, we show how both cost efficiency and performance efficiencies are co-optimized using ACOCA. ACOCA holds the same conclusions we made using Figure 27 and Figure 35 for the 1-SLA scenario in the n-SLA scenario as well.

## 7. Conclusions

In this paper, we introduced an adaptive algorithm for caching context (i.e., ACOCA) along with mathematical models aimed at achieving cost and performance efficiencies. Our ACOCA algorithm is novel in the area of context management since the previous implementation of a context cache was not found in the literature. Context is interpreted data about entities that are different from data that are traditionally discussed with adaptive caching in the literature. For example, we prove that caching the most popular context as in traditional context-aware data caching does not yield the maximum hit rate or the cost efficiency for the CMP, indicative of the nontrivial nature of this problem. Hence, caching context needs to be viewed from a different perspective to data, especially for IoT-based applications that are time critical in nature, which requires context to be derived and delivered to the consumer quickly while being inexpensive. We developed and presented the theories upon which our adaptive context-caching algorithm is developed to maximize both cost and performance efficiencies. We tested our novel algorithm using a large load of context queries and benchmarked it against the redirector mode, traditional cache-all policy, and the context-aware adaptive caching policy developed to maximize the hit rate. ACOCA was integrated into the context-as-a-service platform for evaluation under two scenarios: a single consumer (1-SLA) and multiple context-consumers (n-SLA). The key insights of this paper are as follows:

- We developed a mathematical model for the ACOCA mechanism, focusing on each stage of the lifecycle;
- We developed and tested an ACOCA mechanism that maximized the cost and performance efficiencies of a CMP. The experimental results showed our mechanism reaches a quasi-optimal state that was better than any benchmarks;
- Our novel mechanism was aware of different heterogeneities (e.g., quality of context requirements of context consumers) and incorporated strategies either mathematically or algorithmically to handle them. Hence, ACOCA was tested for complex n-SLA scenarios using a heterogeneous query load. To the best of the authors’ knowledge, it was the first time such an experiment was performed on a context-caching mechanism.
- We proved the inapplicability of traditional caching techniques for caching context information. Traditional context-aware caching policies were shown to incur higher costs compared to ACOCA, proving our theory of the “exploding cost of adaptive context management”.
- We showed that the efficiency benefits of the ACOCA mechanism could be equally derived under dynamic homogeneous (e.g., 1-SLA scenario) or heterogeneous (e.g., n-SLA scenario) context-query loads.

Under the 1-SLA scenario, we first showed that ACOCA is more performance efficient than the benchmarks having recorded the least context-query response time of 2.198 s, and a probability of delay of 0.58, resulting in the highest throughput of 53.8 per minute. The average hit rate was 10% higher than the context-aware policy that attempt to maximize the hit rate. Then, upon investigating the cost efficiency of ACOCA, we revealed that our policy is up to 87%, 56%, and 13% more cost efficient compared to our benchmarks—traditional data caching, redirector mode, and context-aware adaptive data caching, respectively. Finally, we compared the cost of introducing the complexity of adaptation to the CMP versus the earnings from responding to context queries. Out of the four stages in the ACOCA lifecycle, selection, refreshing, scaling, and eviction, we implemented adaptive context selection, refreshing, and eviction in this paper. ACOCA incurred only 99.8% of the processing costs per second in this respect compared to the redirector mode, which was a significant improvement in computing-resource utilization as well.

The n-SLA scenario also produced similar results, as in 1-SLA, indicating the cost and performance-efficiency advantages of ACOCA profoundly against the benchmarks. For instance, ACOCA was up to 68.6%, 84.7%, and 67% more cost efficient compared to traditional data-caching policies to cache context, redirector mode, and context-aware adaptive data caching. We show that the additional complexity introduced by ACOCA is negligible in the n-SLA scenario with heterogeneous context queries as well, providing a solid argument as to why ACOCA is significantly advantageous in real-world settings.

The most-important finding of this work was affirming the concept of the “exploding cost of adaptive context management” which we discovered in theory. The cost of context management was up to 82.9% higher compared to ACOCA in the 1-SLA scenario using traditional caching techniques. By benchmarking ACOCA against a context-aware adaptive caching policy aimed at optimizing the hit rate, and a traditional cache-all (and evict) policy under three context-refreshing algorithms, reactive, proactive, and resolved, we showed that these techniques are not fully applicable to adaptive context caching. The lifecycle costs of context caching were identified as the driving factor for this observation.

As further work, we aim to develop a proactive selective context-caching algorithm by extending the use of the performance monitor and the context query classes to identify and use associative sequences to predict and cache context information.

## Figures and Tables

**Table 1:** Differences between data and context caching.

Feature	Data Caching	Context Caching
Refreshing	Only for transient data.	Necessary.
Fault tolerance	Recoverable from the data provider.	Unrecoverable (need to re-interpret).
Prior knowledge of offline learning	Available (e.g., data libraries, transition probabilities).	Unavailable, uncertain, or limited.
Quality concerns about cached data	Limited (e.g., response latency, hit rate)	Multivariate and complicated.
Size of possible caching actions	Predefined and limited. often based on the number of distinct data.	Evolving and cannot be predefined at all.

**Table 2:** Notations used in the paper.

Notation	Description	Notation	Description
*i*	Index of a piece of context information in I, i.e., *i*$\in \left\{1,2,\ldots I\right\}$.	Earnings	The monetary earnings received from responding to context queries while adhering to the quality parameters.
*n*	Index of an applicable Service Level Agreement in N, i.e., *n*$\in \left\{1,2,\ldots N\right\}$.	$Cost_{process}$	The cost of processing for the context.
M	Number of context providers from which the data is retrieved to infer a piece of context information.	$Cost_{penalty}$	The cost incurred as penalties due to nonadherence to quality parameters when responding to context queries.
C	Number of related contexts of higher logical order.	$Cost_{storage}$	The cost of storing the context in persistent storage.
HR	Hit Rate, where 0≤HR≤1.	$Cost_{cache}$	Total cost incurred to store in the cache.
MR	Miss Rate, where 0≤MR≤1.	$Cost_{retrieval}$ or $Cost_{ret}$	Cost of retrieving a context data.
PD	Probability of Delay, where 0≤PD≤1 [11].	$Cost_{redir}$	Cost of responding to context queries using the redirector mode.
λ	Request rate, e.g., 1 per second.	$Cost_{cached}$	Cost of managing a context in cache.
$f_{thr}$	Freshness threshold—the minimum freshness tolerated by a Context Consumer when requesting a piece of context information.	$Cost_{caching}$	Cost of caching a physical unit in the cache, e.g., AUD 0.3 per Giga Byte.
AR	Access Rate, e.g., 0.8 per second.	W	Size of a window, e.g., 60 s.
ExpPrd	Expiry Period—the time period during which a context is considered fresh enough to be used in responding to a context query [11,12].	$OH_{partialmiss}$	The cache-lookup overhead when a partial miss occurs.
PP	Planning Period [12].	$OH_{hit}$	The cache-lookup and retrieval overhead when a cache hit occurs.
IR	Invalid Rate, where 0≤IR≤1 [11].	$OH_{fullmiss}$	The cache-lookup overhead when a complete (i.e., full) miss occurs.
*InvGap*	Invalid Gap [11].	$Cost_{\left(ret|redir\right)}$	The cost of context retrieval for the CMP when using the redirector mode.
*InvPrd*	The time until the subsequent retrieval from the point of time the freshness threshold is no longer met.	$Cost_{(ret|cached)\;}$	The cost of context retrieval from the CMP with context caching.
*AcsInt*	The average time between two requests for the same context, which is equal to 1/AR.	RetCost	The cost to retrieve the context data.
$\bar{Gain}$	Average monetary gain from responding to any context query.	PenaltyCost	The cost to be incurred as penalties for not meeting the quality parameters set by the context consumer.
$t_{g}$	The gap time between the time a context is expired and refreshed.	$RT_{max}$	The expected maximum accepted response latency for the context consumers.
$P\left(f_{thr,n}\right)$	The probability of *f_thr_* from the n^th^ SLA being applied on an accessed context information.	*RR*	Refresh Rate, e.g., 0.5 per second.
$\sigma^{2}$	Variance of a distribution.	$En_{x}$	Context entity.
$N_{exp}$	The number of expensive SLAs.	$a_{x}$	Context attribute.
L	Lifetime of a context.	CL	Estimated cache lifetime (i.e., residence time).
SI	Sampling Interval of a sensor.	DT	Estimated delay time.
RetL	Context retrieval latency.	$Cost_{ref}$	Cost of refreshing a context.
ResiL	Residual Lifetime.	nonRetEff	The sum of weighted parameters other than $E\left[RE\right]$ where $conf\left(i\right)=0$.
age	Age of context information.	$Z_{\theta }$	Calculated z-value for the estimated θ.
$Cost_{ret}$	Cost of context retrieval.	$conf\left(i\right)$	Confidence to selectively cache *i*.
$TotalCost_{ret}$	$\mathrm{Total}\;\cos t\;\mathrm{of}\;\mathrm{context}\;\mathrm{retrieval}\;\mathrm{during}\;a\;t_{g}$.	CE	Cache Efficiency.
K	Number of gaps, where K < N-1.	RE	Retrieval Efficiency.
S	The number of successful retrievals.	AT	Access Trend.
R	The total number of context retrievals attempted.	Unreli	Unreliability of context retrieval for a piece of context, i.e., *Unreli = 1–Reliability*.
confL	Confidence of the inferred lifetime.	Cmpx	The complexity of a context query.
$\bar{Gain}$.	Average monetary gain per context query from responding to context queries.	Cmpx(i)	The probabilistic complexity of context queries that would access the context information *i*.
$\bar{Conf}$	Average of the historical $conf\left(i\right)$ sample.	κ,μ,ω,ρ,δ	Weights that are assigned to each of the parameters in the $conf\left(i\right)$ formula.
$v\left(i\right)$	The feature vector of a candidate context *i* to cache.	ϑ	Caching decision threshold.
φ	Set of all the weights.	θ	Cache distribution bias, where 0≤θ≤1.
AUD	Australian Dollar.	$\sigma_{conf}$	The standard deviation of the sample of $conf\left(i\right)$ values.

**Table 3:** Conditions of selecting the refreshing policy.

Data Ingestion	CP Sampling	L < SI?	Refreshing Policy
Streamed	Periodic	False	Depends on *ExpPrd*
		True	Reactive
Fetched	Aperiodic	False	Proactive
		True	
	Periodic	False	Depends on *ExpPrd*
		True	Reactive

**Table 4:** Complexity of the context queries.

Parameter	Query 1	Query 2	Query 3
$N\left(\left\{Operators\right\}\right)$	4	7	9
$N\left(\left\{Operands\right\}\right)$	6	16	23
$N\left(Operators\right)$	5	14	23
$N\left(Operands\right)$	6	16	25
*Cmpx*	$\frac{4}{2}\times \frac{6}{6}=2$	$\frac{7}{2}\times \frac{16}{16}=3.5$	$\frac{9}{2}\times \frac{25}{23}=4.89$

**Table 5:** Summary of important QoS parameters in Context Consumer SLAs.

	$Price_{res}$	$f_{thr}$	$RT_{max}$	$Cost_{pen}$
Average	AUD 0.5198	0.74	2217 ms	AUD 0.6925
Std Deviation	0.3844	0.1071	618.8027	0.4347
Minimum	AUD 0.0070	0.5	14400 ms	AUD 0.1000
Maximum	AUD 1.4000	0.9	3600 ms	AUD 2.0000

**Table 6:** Summary of cost and performance of the CMP under the 1-SLA scenario.

Parameter	$\bar{Gain}$	$\bar{RT}$	$\bar{PD}$
**ACOCA**	−0.87±0.01	2198.00	0.58±0.01
Context Aware	−1.00±0.02	2576.47	0.65±0.01
CA Reactive	−1.00±0.09	2391.19	0.63±0.04
CA Proactive	−6.60±1.43	3340.00	0.71±0.03
CA Resolved	−5.09±1.05	3084.44	0.71±0.04
Redirector	−1.96±0.01	2894.20	0.98±0.00

**Table 7:** Summary of cost and performance of the CMP under the n-SLA scenario.

Parameter	$\bar{Gain}$	$\bar{RT}$	$\bar{PD}$
**ACOCA**	−0.12±0.01	1844.30±7.06	0.2966
Context Aware	−0.36±0.01	1852.04±9.28	0.3046
CA Reactive	−0.14±0.01	1854.96±11.05	0.2984
CA Proactive	−0.89±0.14	2963.71±367.1	0.4353
CA Resolved	−2.42±0.06	11548.55±292.6	1000
Redirector	−0.81±0.01	3326.12±8.72	0.9176

## Data Availability

The data presented in this study are available in Weerasinghe, S.; Zaslavsky, A.; Hassani, A.; Loke, S.W.; Medvedev, A.; Abken, A. Context Query Simulation for Smart Carparking Scenarios in the Melbourne CDB. *arXiv* **2022**, arXiv:2302.07190.

## References

[1] Perera C., Zaslavsky A., Christen P., Georgakopoulos D. (2014). Context Aware Computing for The Internet of Things: A Survey. IEEE Commun. Surv. Tutor..

[2] Abowd G.D., Dey A.K., Brown P.J., Davies N., Smith M., Steggles P., Gellersen H.-W. (1999). Towards a Better Understanding of Context and Context-Awareness. Handheld and Ubiquitous Computing.

[3] Ruggeri G., Amadeo M., Campolo C., Molinaro A., Iera A. (2021). Caching Popular Transient IoT Contents in an SDN-Based Edge Infrastructure. IEEE Trans. Netw. Serv. Manag..

[4] Liu X., Derakhshani M., Lambotharan S. (2021). Contextual Learning for Content Caching With Unknown Time-Varying Popularity Profiles via Incremental Clustering. IEEE Trans. Commun..

[5] Peng T., Wang H., Liang C., Dong P., Wei Y., Yu J., Zhang L. (2021). Value-aware Cache Replacement in Edge Networks for Internet of Things. Trans. Emerg. Telecommun. Technol..

[6] Jagarlamudi K.S., Zaslavsky A., Loke S.W., Hassani A., Medvedev A. Quality and Cost Aware Service Selection in IoT-Context Management Platforms. Proceedings of the 2021 IEEE International Conferences on Internet of Things (iThings) and IEEE Green Computing & Communications (GreenCom) and IEEE Cyber, Physical & Social Computing (CPSCom) and IEEE Smart Data (SmartData) and IEEE Congress on Cybermatics (Cybermatics).

[7] Hassani A., Medvedev A., Haghighi P.D., Ling S., Indrawan-Santiago M., Zaslavsky A., Jayaraman P.P. Context-as-a-Service Platform: Exchange and Share Context in an IoT Ecosystem. Proceedings of the 2018 IEEE International Conference on Pervasive Computing and Communications Workshops (PerCom Workshops).

[8] Lehmann O., Bauer M., Becker C., Nicklas D. From Home to World—Supporting Context-Aware Applications through World Models. Proceedings of the Second IEEE Annual Conference on Pervasive Computing and Communications.

[9] FIWARE-Orion. https://github.com/telefonicaid/fiware-orion.

[10] Weerasinghe S., Zaslavsky A., Loke S.W., Medvedev A., Abken A., Hassani A. (2023). Context Caching for IoT-based Applications: Opportunities and Challenges. IEEE Internet Things J..

[11] Weerasinghe S., Zaslavsky A., Loke S.W., Medvedev A., Abken A. Estimating the Lifetime of Transient Context for Adaptive Caching in IoT Applications. Proceedings of the ACM Symposium on Applied Computing.

[12] Medvedev A. (2020). Performance and Cost Driven Data Storage and Processing for IoT Context Management Platforms. Doctoral Thesis.

[13] Sheng S., Chen P., Chen Z., Wu L., Jiang H. Edge Caching for IoT Transient Data Using Deep Reinforcement Learning. Proceedings of the IECON 2020 The 46th Annual Conference of the IEEE Industrial Electronics Society.

[14] Zhang Z., Lung C.-H., Lambadaris I., St-Hilaire M. IoT Data Lifetime-Based Cooperative Caching Scheme for ICN-IoT Networks. Proceedings of the 2018 IEEE International Conference on Communications (ICC).

[15] Weerasinghe S., Zaslavsky A., Loke S.W., Hassani A., Abken A., Medvedev A. (2022). From Traditional Adaptive Data Caching to Adaptive Context Caching: A Survey. arXiv.

[16] Boytsov A., Zaslavsky A. From Sensory Data to Situation Awareness: Enhanced Context Spaces Theory Approach. Proceedings of the 2011 IEEE Ninth International Conference on Dependable, Autonomic and Secure Computing.

[17] Sun Y., Uysal-Biyikoglu E., Yates R., Koksal C.E., Shroff N.B. Update or Wait: How to Keep Your Data Fresh. Proceedings of the IEEE INFOCOM 2016—The 35th Annual IEEE International Conference on Computer Communications.

[18] Schwefel H.-P., Hansen M.B., Olsen R.L. Adaptive Caching Strategies for Context Management Systems. Proceedings of the 2007 IEEE 18th International Symposium on Personal, Indoor and Mobile Radio Communications.

[19] Zameel A., Najmuldeen M., Gormus S. Context-Aware Caching in Wireless IoT Networks. Proceedings of the 2019 11th International Conference on Electrical and Electronics Engineering (ELECO).

[20] Li Q., Shi W., Xiao Y., Ge X., Pandharipande A. Content Size-Aware Edge Caching: A Size-Weighted Popularity-Based Approach. Proceedings of the 2018 IEEE Global Communications Conference (GLOBECOM).

[21] Weerasinghe S., Zaslavsky A., Loke S.W., Abken A., Hassani A., Medvedev A. Adaptive Context Caching for Efficient Distributed Context Management Systems. Proceedings of the ACM Symposium on Applied Computing.

[22] Cidon A., Eisenman A., Alizadeh M., Katti S. Cliffhanger: Scaling Performance Cliffs in Web Memory Caches. Proceedings of the NSDI’16: Proceedings of the 13th Usenix Conference on Networked Systems Design and Implementation.

[23] Arcaini P., Riccobene E., Scandurra P. Modeling and Analyzing MAPE-K Feedback Loops for Self-Adaptation. Proceedings of the 2015 IEEE/ACM 10th International Symposium on Software Engineering for Adaptive and Self-Managing Systems.

[24] Fizza K., Banerjee A., Jayaraman P.P., Auluck N., Ranjan R., Mitra K., Georgakopoulos D. (2022). A Survey on Evaluating the Quality of Autonomic Internet of Things Applications. IEEE Commun. Surv. Tutor..

[25] Wang Y., He S., Fan X., Xu C., Sun X.-H. (2019). On Cost-Driven Collaborative Data Caching: A New Model Approach. IEEE Trans. Parallel Distrib. Syst..

[26] Zhu H., Cao Y., Wei X., Wang W., Jiang T., Jin S. (2019). Caching Transient Data for Internet of Things: A Deep Reinforcement Learning Approach. IEEE Internet Things J..

[27] Khargharia H.S., Jayaraman P.P., Banerjee A., Zaslavsky A., Hassani A., Abken A., Kumar A. Probabilistic Analysis of Context Caching in Internet of Things Applications. Proceedings of the 2022 IEEE International Conference on Services Computing (SCC).

[28] Kiani S., Anjum A., Antonopoulos N., Munir K., McClatchey R. (2012). Context Caches in the Clouds. J. Cloud Comput. Adv. Syst. Appl..

[29] Wang Y., Friderikos V. (2020). A Survey of Deep Learning for Data Caching in Edge Network. Informatics.

[30] Shuja J., Bilal K., Alasmary W., Sinky H., Alanazi E. (2021). Applying Machine Learning Techniques for Caching in Next-Generation Edge Networks: A Comprehensive Survey. J. Netw. Comput. Appl..

[31] Guo Y., Lama P., Rao J., Zhou X. V-Cache: Towards Flexible Resource Provisioning for Multi-Tier Applications in IaaS Clouds. Proceedings of the 2013 IEEE 27th International Symposium on Parallel and Distributed Processing.

[32] Garetto M., Leonardi E., Martina V. (2016). A Unified Approach to the Performance Analysis of Caching Systems. ACM Trans. Model. Perform. Eval. Comput. Syst..

[33] Sadeghi A., Wang G., Giannakis G.B. (2019). Deep Reinforcement Learning for Adaptive Caching in Hierarchical Content Delivery Networks. IEEE Trans. Cogn. Commun. Netw..

[34] Al-Turjman F., Imran M., Vasilakos A. (2017). Value-Based Caching in Information-Centric Wireless Body Area Networks. Sensors.

[35] Somuyiwa S.O., Gyorgy A., Gunduz D. (2018). A Reinforcement-Learning Approach to Proactive Caching in Wireless Networks. IEEE J. Select. Areas Commun..

[36] Nasehzadeh A., Wang P. A Deep Reinforcement Learning-Based Caching Strategy for Internet of Things. Proceedings of the 2020 IEEE/CIC International Conference on Communications in China (ICCC).

[37] Al-Turjman F.M., Al-Fagih A.E., Hassanein H.S. A Value-Based Cache Replacement Approach for Information-Centric Networks. Proceedings of the 38th Annual IEEE Conference on Local Computer Networks—Workshops.

[38] Weerasinghe S., Zaslavsky A., Loke S.W., Abken A., Hassani A. (2022). Reinforcement Learning Based Approaches to Adaptive Context Caching in Distributed Context Management Systems. arXiv.

[39] Medvedev A., Zaslavsky A., Indrawan-Santiago M., Haghighi P.D., Hassani A., Galinina O., Balandin S., Koucheryavy Y. (2016). Storing and Indexing IoT Context for Smart City Applications. Internet of Things, Smart Spaces, and Next Generation Networks and Systems.

[40] FIWARE-Orion Components. https://www.fiware.org/catalogue/.

[41] Jung J., Berger A.W. Hari Balakrishnan Modeling TTL-Based Internet Caches. Proceedings of the IEEE INFOCOM 2003. Twenty-second Annual Joint Conference of the IEEE Computer and Communications Societies (IEEE Cat. No.03CH37428).

[42] Larson R.C., Odoni A.R. (2007). Urban Operations Research.

[43] Weerasinghe S., Zaslavsky A., Loke S.W., Medvedev A., Abken A. (2022). Estimating the Dynamic Lifetime of Transient Context in near Real-Time for Cost-Efficient Adaptive Caching. SIGAPP Appl. Comput. Rev..

[44] Lillicrap T.P., Hunt J.J., Pritzel A., Heess N., Erez T., Tassa Y., Silver D., Wierstra D. (2019). Continuous Control with Deep Reinforcement Learning. arXiv.

[45] Appendix to Adaptive Context Caching for IoT-Based Applications. https://bit.ly/3eEMJxc.

[46] Fujimoto S. Addressing Function Approximation Error in Actor-Critic Methods. Proceedings of the 35th International Conference on Machine Learning.

[47] Wu X., Li X., Li J., Ching P.C., Leung V.C.M., Poor H.V. (2021). Caching Transient Content for IoT Sensing: Multi-Agent Soft Actor-Critic. IEEE Trans. Commun..

[48] Hassani A., Medvedev A., Delir Haghighi P., Ling S., Zaslavsky A., Prakash Jayaraman P. (2019). Context Definition and Query Language: Conceptual Specification, Implementation, and Evaluation. Sensors.

[49] Kul G., Luong D.T.A., Xie T., Chandola V., Kennedy O., Upadhyaya S. (2018). Similarity Metrics for SQL Query Clustering. IEEE Trans. Knowl. Data Eng..

[50] Kul G., Luong D., Xie T., Coonan P., Chandola V., Kennedy O., Upadhyaya S. Ettu: Analyzing Query Intents in Corporate Databases. Proceedings of the 25th International Conference Companion on World Wide Web—WWW ’16 Companion.

[51] Yang J., McAuley J., Leskovec J., LePendu P., Shah N. Finding Progression Stages in Time-Evolving Event Sequences. Proceedings of the 23rd international conference on World wide web—WWW ’14.

[52] Sheikh R., Kharbutli M. Improving Cache Performance by Combining Cost-Sensitivity and Locality Principles in Cache Replacement Algorithms. Proceedings of the 2010 IEEE International Conference on Computer Design.

[53] Weerasinghe S., Zaslavsky A., Hassani A., Loke S.W., Medvedev A., Abken A. (2022). Context Query Simulation for Smart Carparking Scenarios in the Melbourne CDB. arXiv.

